# Unconventional application of the Mitsunobu reaction: Selective flavonolignan dehydration yielding hydnocarpins

**DOI:** 10.3762/bjoc.12.66

**Published:** 2016-04-08

**Authors:** Guozheng Huang, Simon Schramm, Jörg Heilmann, David Biedermann, Vladimír Křen, Michael Decker

**Affiliations:** 1 Pharmazeutische und Medizinische Chemie, Institut für Pharmazie und Lebensmittelchemie, Julius-Maximilians-Universität Würzburg, Am Hubland, D-97074 Würzburg, Germany; 2 College of Life and Environmental Sciences, Shanghai Normal University, Shanghai, P. R. China; 3 Lehrstuhl für Pharmazeutische Biologie, Institut für Pharmazie, Universität Regensburg, Universitätsstraße 31, D-93053 Regensburg, Germany; 4 Centre of Biotransformation and Biocatalysis, Institute of Microbiology, Czech Academy of Sciences, Videnska 1083, Prague 4, CZ-14220, Czech Republic

**Keywords:** dehydration, flavonoid, hydnocarpin, Mitsunobu, silybin

## Abstract

Various Mitsunobu conditions were investigated for a series of flavonolignans (silybin A, silybin B, isosilybin A, and silychristin A) to achieve either selective esterification in position C-23 or dehydration in a one-pot reaction yielding the biologically important enantiomers of hydnocarpin D, hydnocarpin and isohydnocarpin, respectively. This represents the only one-pot semi-synthetic method to access these flavonolignans in high yields.

## Introduction

**Figure 1:**
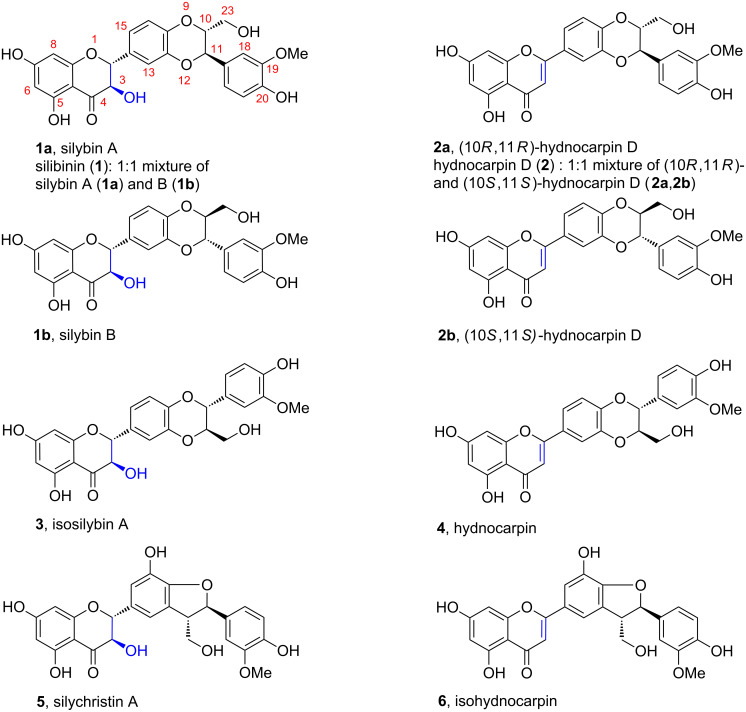
Structures of silibinin, isosilybin, and silychristin, and hydnocarpin-type flavonolignans.

Flavonolignans combine the structural moieties of flavonoids and phenylpropanoids (lignans). Among the group of flavonolignans, the medicinally most important ones can be found in *Silybum marianum* (L.) Gaertn., the milk thistle*.* The phenolic secondary metabolites of this plant have been intensively studied due to their multiple biological activities and promising therapeutic applications, such as hepatoprotection, antitumor, antiproliferative and anti-oxidant properties [1]. The standardized extract of *S. marianum* fruits contains the so-called silymarin complex, and is used as the main active component mixture of Legalon^®^, a drug approved for treatment of chronic hepatitis, hepatoprotection in alcohol addicts [2], or intoxication with *Amanita phalloides*, the death cap [3]. Silibinin (**1**, Figure 1), the major component of silymarin, became the first isolated flavonolignan [4]. Besides **1**, this extract consists also of isosilibinin (**3**), silychristin (**5**), silydianin (**11**), and dehydrosilibinin [1,5]. Natural and commercially available silibinin is the diastereomeric mixture (quasi equimolar) of silybin A (**1a**) and silybin B (**1b**).

Because of the well-known biological properties of silibinin and the other compounds of the silymarin complex for protection of liver damage, and for preventing skin tumor promotion, these compounds have attracted efforts on their total synthesis (e.g., isosilybin) [6] and structural modification [5,7–9] to improve their water-solubility, bioavailability and biological activities. Recently, our group has developed a hybrid drug combining silibinin with tacrine (a potent acetylcholinesterase inhibitor for treatment of Alzheimer’s disease), which shows neuro- and hepatoprotective effects exceeding the cytoprotective effects of silibinin and effectively counteracts tacrine’s dose-dependent hepatotoxicity in vitro as well as in vivo [10–11].

The flavonolignan hydnocarpin (**4**, Figure 1) was first isolated in 1973 from *Hydnocarpus wightiana* [12], and its structure was at first assigned erroneously as what is now named hydnocarpin D (**2**, Figure 1) (for details see below) [13]. The actual hydnocarpin D was first synthesized [14] and later isolated from various plant species (for reviews see [5,15]). In several references the nomenclature of hydnocarpins, assignment of their structures and/or enantiomeric purities are not used coherently or experimental and full structural evidence have not been provided [15].

Hydnocarpin-type compounds (**2**, **4** and **6**; Figure 1) are formally dehydrated analogs of silymarin flavonolignans with flavanone-3-ol (3-hydroxyflavanone) structure (silybin A and B, isosilybin A and silychristin A). Hydnocarpin and its derivatives show interesting biological activities such as being efficient inhibitors of the multidrug resistance (MDR) efflux pump (e.g., of *Staphylococcus aureus*): this activity had been serendipiously employed in traditional medicine (without knowledge of the mechanism) in the treatment of leprosy with chaulmoogra oil. This oil, which is obtained from fruits (kernels) of *H. wightiana*, contains besides hydnocarpin mainly cyclopentenoic fatty acids [16], which show pronounced antibiotic activity and inhibit multiplication of mycobacteria [17]. Combination of these antibiotics together with hydnocarpin (MDR inhibitor) helped to treat such a persistent disease like leprosy caused by *M. leprae*. Recently, hydnocarpin (isolated from *Brucea javanica*; which was named as (−)-hydnocarpin, even though the enantiomeric purity was not determined) has been described as potentiator of vincristine’s cytotoxicity due to MDR inhibition [18]. Furthermore, 5-methoxyhydnocarpin has been described to enhance the antimicrobial activity of berberine [19]. In the light of steadily growing antibiotic resistance any potent and nontoxic MDR inhibitor is of utmost importance. Hydnocarpin has also antineoplastic activity due to sensitizing multidrug-resistant cancer cell lines towards chemotherapy and no toxicity was found in vivo [20].

**Figure 2:**
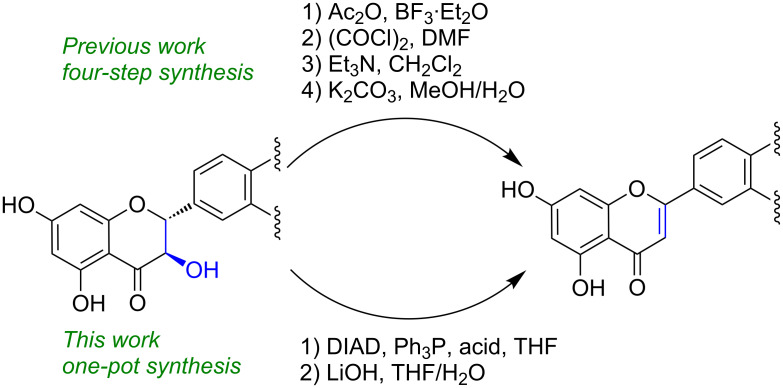
Synthetic strategy of semi-synthesis of hydnocarpins from silybins [22].

Unlike for silybins [6], there are only a few examples of total syntheses or even structural modifications of hydnocarpin-type (flavone) flavonolignans – simply because they are not synthetically available [14,21]. A straightforward synthetic approach such as a semi-synthetic one from readily available material will provide access to this class of highly interesting natural products and derivatives with putatively differing biological activities. A very recent example of conversion of silybins to hydnocarpins was described by Vimberg et al., who applied a four-step synthesis and used Vilsmeier–Haack conditions (Figure 2) [22]. Before this, the only reference for the synthetic preparation of hydnocarpin D used coniferyl alcohol and luteolin as starting materials, and obtained hydnocarpin D as a minor product only (7%, catalyzed by Ag_2_CO_3_; 5% catalyzed by horseradish peroxidase) [14].

To study the biological activities of hydnocarpin-type compounds, a new and robust method for their preparation is required. It is known that elimination can occur in some cases as a side reaction in Mitsunobu esterifications with structurally hindered alcohols and bulky acids: Cherney and Wang observed an elimination of a serine derivative during esterification experiments [23]. During conversion of substituted benzyl alcohols to amines under Mitsunobu conditions, elimination also occurred [24]. Dehydration under Mitsunobu conditions has been applied in modifications of natural products [23–24], such as *N*-Boc neomycin B under epoxide formation [25]. Elson et al. systematically investigated the mechanism of the reaction of menthol with *p*-nitrobenzoic acid using a Hendrickson or Mitsunobu reagent [26]. The Hendrickson reagent led to conversion of menthol into the β-elimination product. In all the above cases the substrate structures are very different from flavanone-3-ols, but since the 3-OH group of the silymarin flavonolignans is also hindered, we have envisioned that Mitsunobu conditions might enable the conversion of silybins to hydnocarpins. Herein, we summarize our achievements on converting silibinin (including the two isolated diasteromers silybin A and silybin B) to hydnocarpin D (and its two enantiomers) under various Mitsunobu reaction conditions, and applying these conditions to the other silymarin congeners, which provided an effective and expeditious semi-synthetic preparation of hydnocarpin, hydnocarpin D and isohydnocarpin.

## Results and Discussion

Although silibinin, i.e., silybins A and B, bears three phenolic, one secondary and one primary hydroxy group, selective Mitsunobu esterification typically occurs at the primary C-23 OH (position 11 according to IUPAC nomenclature, but rarely used in the literature) without attacking other hydroxy groups, as reported by Wang et al. [9], and by our group [10]. Two different sequences of addition of reactants were applied, i.e., addition of solution of acid to the mixture of silibinin, triphenylphosphine (Ph_3_P) and diethyl azodicarboxylate (DEAD) [9], versus addition of diisopropyl azodicarboxylate (DIAD) to the mixture of silibinin, acid and Ph_3_P [10]. In both cases, the desired C-23 ester was obtained in moderate yields, and we observed a side reaction, which we expected to be a dehydration reaction.

**Scheme 1:**
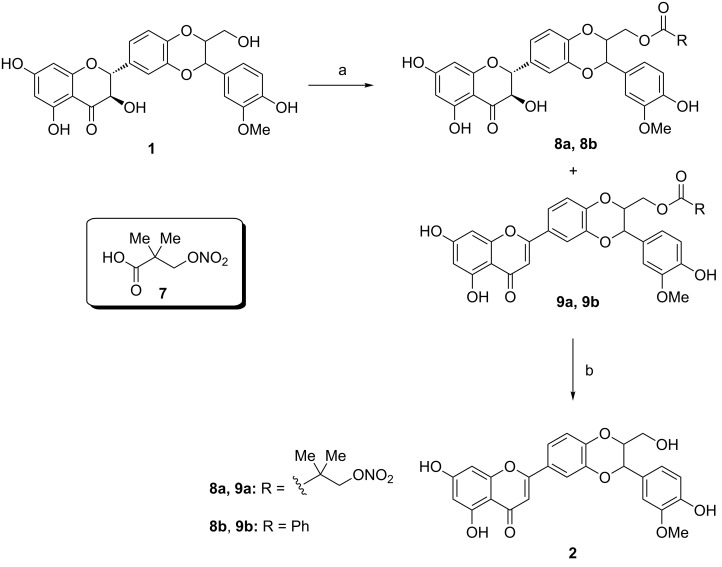
Synthesis of ester derivatives of silibinin and conversion to hydnocarpin-type compounds. Reaction conditions: a) **7** or benzoic acid, Ph_3_P, diisopropyl azodicarboxylate (DIAD), THF, 10 °C to rt, 3 h.; b) LiOH·H_2_O, MeOH/H_2_O, rt, 2 h.

We investigated the reaction under Mitsunobu conditions using the latter sequence of addition [10] for silibinin and benzoic acid, or 2,2-dimethyl-3-(nitrooxy)propanoic acid (**7**, Scheme 1), an NO-donor compound with several biological properties, e.g., vasorelaxation [27]. Compound **7** had initially been used by us since we wanted to obtain hybrid esters for medicinal chemical purposes [27]. As expected, in all cases, two products were detected in different ratios according to the different ratios of Ph_3_P and DIAD used (Table 1, entries 1–4). Spectral data of the two products confirmed their structures as esters of silibinin (major product, **8a**, **8b**) and hydnocarpin D (minor product, **9a**, **9b**). 2,3-Olefination was further proven by hydrolysis of compound **9a**, which converted the esters into the racemic mixture of (10*R*,11*R*)- and (10*S*,11*S*)-hydnocarpin D (enantiomers **2a** and **2b**). We then systematically investigated dehydration by varying the amounts of Ph_3_P and DIAD, respectively (Table 1). It was observed that excessive Ph_3_P and DIAD shifted the ratio of products toward the desired hydnocarpin D esters, while using a lower amount of Mitsunobu reagents favored the silibinin ester formation (Table 1, entries 1 and 5). In absence of an acid, 2,3-dehydration did not occur even using excessive amounts of Ph_3_P and DIAD (Table 1, entries 7 and 8). In the absence of DIAD, silibinin was not converted into any other compound (Table 1, entry 9). Benzoic acid had been applied to synthesize 23-*O*-benzoylsilibinin (**8b**) in 55% yield [9]. Using benzoic acid with 6 equiv of Ph_3_P and 4 equiv of DIAD, silibinin was converted into ester **9b** with a yield of 33% after purification (Table 1, entry 4). Again hydrolysis of **9b** yielded hydnocarpin D (**2**).

**Table 1:** Reaction conditions for optimization of dehydration.

	Reaction conditions			Products
	Ph_3_P	DIAD	Acid	

1	2.5 equiv	2.5 equiv	**7**, 2.0 equiv	**8a** and **9a** (39% and 6%)^a^
2	6.0 equiv	3.0 equiv	**7**, 3.0 equiv	**8a** and **9a** (11:41)^b^
3	6.0 equiv	3.0 equiv	PhCO_2_H, 3.0 equiv	**8b** and **9b** (19:81)^b^
4	6.0 equiv	4.0 equiv	PhCO_2_H, 3.0 equiv	**9b** (33%)^a^
5	4.0 equiv	2.0 equiv	**7**, 2.0 equiv	**8a** (82%)^a^
6	6.0 equiv	4.0 equiv	**7**, 3.0 equiv	**9a** (40%)^a^
7	2.0 equiv	2.0 equiv	–	no reaction
8	6.0 equiv	4.0 equiv	–	no reaction
9	6.0 equiv	–	**7**, 3.0 equiv	no reaction

^a^Isolated yield. ^b^Ratio by HPLC, see Figures S1 and S2 in Supporting Information File 1.

Being encouraged by the feasibility of using a common acid but still disappointed by the yields, we carefully monitored the process of the reaction and found that ester **8b** was initially formed, and it was then gradually converted into **9b** by the addition of excess DIAD. In the course of the reaction, less polar new products were also formed, which we assumed to be the oligo-esters. After total ester hydrolysis (of both mono- and oligo-esters) the hydnocarpin D-type entities formed, therefore it was not necessary to isolate the monoester prior to hydrolysis. We chose *p*-nitrobenzoic acid to accomplish the reaction, since the corresponding esters are readily hydrolyzed [28]. A Mitsunobu reaction was conducted at higher temperature to prepare hydnocarpin D, and without separation of the different esters formed the crude mixture was saponified in a one-pot way, further improving the (overall) yield to 56%.

Our method is robust, simple and the starting material (silibinin) is cheap and readily available. As mentioned above, natural silibinin represents a mixture of two diastereomers, therefore the dehydration product is also composed of two stereomers (enantiomers). The use of diastereomerically pure compounds (silybin A and silybin B, each of them with a de of 94%), therefore yielded the individual enantiomers (10*R*,11*R*)- and (10*S*,11*S*)-hydnocarpin D (**2a**, **2b**). We measured optical rotations and ECD and confirmed the proposed structures (see Figures S3 and S4 in Supporting Information File 1). To our knowledge, this is the second case only [22] that these two enantiomers were obtained as pure and fully characterized compounds, namely by chiroptical methods. It should be mentioned that previous studies on hydnocarpin D failed to determine its absolute configuration. In 1973 Ranganathan and Seshadri isolated hydnocarpin D from *H. wightiana* for the first time and determined the stereochemistry of the substituents at C-10 and C-11 [12]. However, neither in this nor in later papers on hydnocarpin D the absolute configuration had been properly established. Also Guz et al. denote their compound as (±)-hydnocarpin D, which means that no optically pure compound was obtained [14].

**Figure 3:**
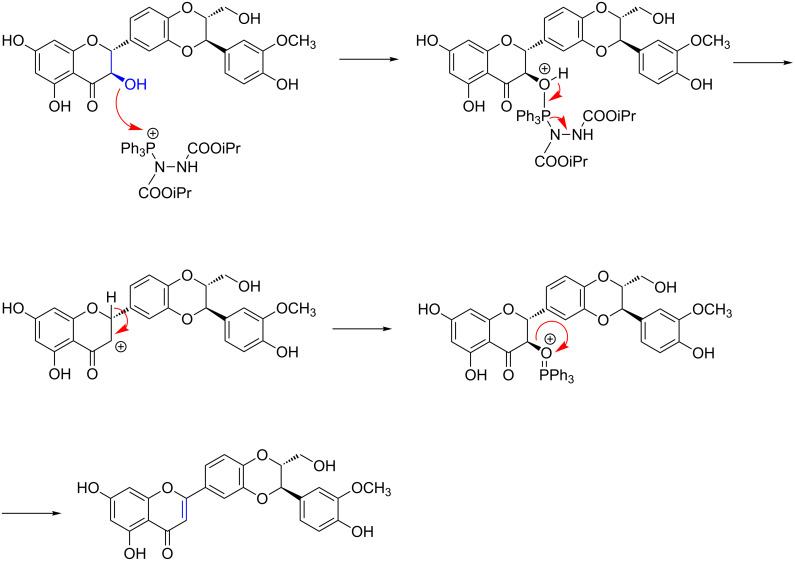
Putative mechanism of dehydration of flavanonols under Mitsunobu conditions.

The Mitsunobu reaction represents a powerful method to convert primary and secondary alcohols into ester but also into various derivatives. The mechanism is well described and includes the formation of the triphenylphosphine–DIAD adduct, which then activates the alcohol making it a good leaving group susceptible to a nucleophilic attack. Application of the Mitsunobu reaction for dehydration was also reported previously as shown in the above-mentioned cases [23–26]. The ratio between possible substitution and elimination products could not be determined in our experiments, but conditions are described now leading to the elimination product. Figure 3 shows a putative reaction mechanism leading to the formation of the eliminated product.

**Scheme 2:**
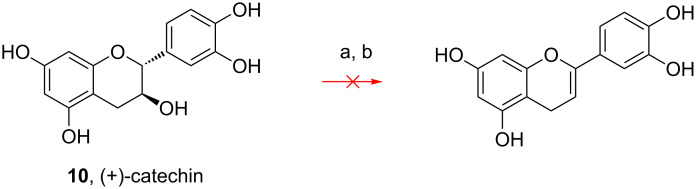
Attempt to dehydrate catechin. Reagents and conditions: a) *p*-nitrobenzoic acid, Ph_3_P, DIAD, THF, rt, 20 h; b) 2 N NaOH, rt, 1 h.

As (+)-catechin (**10**, Scheme 2) cannot produce the desired dehydrated compound under our conditions, we assume the carbonyl group is necessary in the reaction described here, since then β-elimination generates a thermodynamically more stable compound (Scheme 2). Therefore, flavanones dehydration under Mitsunobu conditions requires a 3-hydroxyflavanone (flavanonol) structure.

**Scheme 3:**
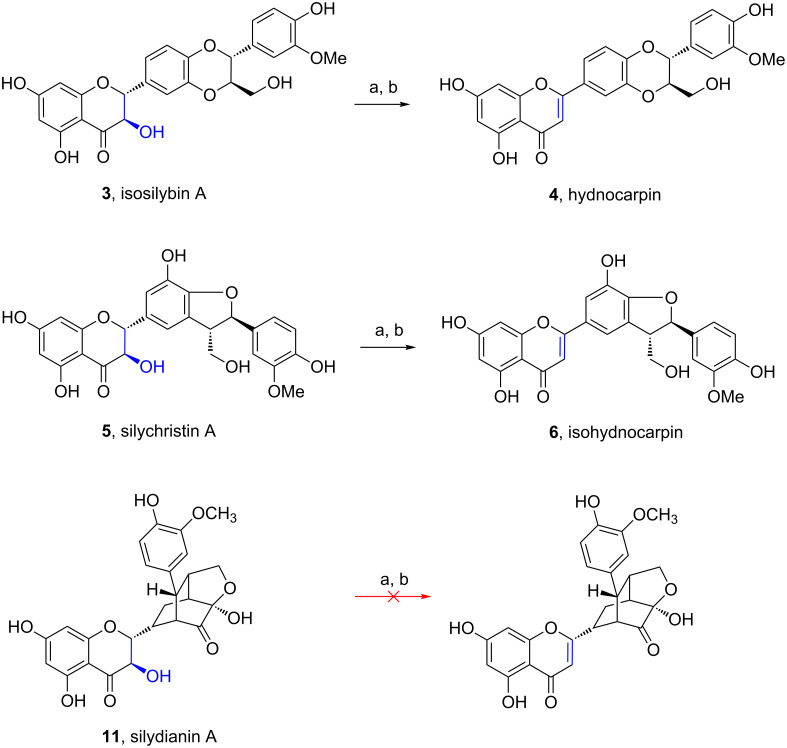
Preparation of hydnocarpin (**4**) and isohydnocarpin (**6**) and attempt to dehydrate silydianin A (**11**). Reagents and conditions: a) *p*-nitrobenzoic acid, Ph_3_P, DIAD, THF, rt, 20 h; b) 2 N NaOH, rt, 1 h.

Accordingly, we converted other 3-hydroxyflavanones into their dehydrated analogs: isosilybin A (containing typically 5% of the B isomer), and silychristin A yielded hydnocarpin (24%), and isohydnocarpin (22%), respectively. This demonstrates the broad applicability of this method and provides until now the only semi-synthetic preparation of these flavonolignans (Scheme 3). Therefore, our method provides a simple and robust semi-synthetic access to all hydnocarpins with pronounced biological activities. When applying the optimized Mitsunobu conditions for elimination of 3-hydroxyflavanone to silydianin (**11**, Scheme 3), another major component of the silymarin complex, decomposition was observed yielding several products (Scheme 3). Since the cyclic hemiacetal structure (of a diketone) represents the only functional difference to the other flavanonols employed, it is obviously unstable under the conditions used.

## Conclusion

To our knowledge, this is the first semi-synthesis of optically pure (10*R*,11*R*)- and (10*S*,11*S*)-hydnocarpin D described to date and gives 56% yield starting from commercially available silibinin in a one-pot reaction. Evaluation of Mitsunobu conditions and reagents applied for esterification and dehydration, respectively, enabled us to exclusively obtain either the hydnocarpin compound esters (and therefore hydnocarpin-type compounds after hydrolysis) or esterification. The recently reported method by Vimberg et al. [22], that was published during the preparation of this article, describes a four-step synthesis using Vilsmeier–Haack conditions. Our study exhibits good atom economics and remains the only specific application of Mitsunobu conditions for a one-pot dehydration of this important class of natural products.

## Supporting Information

File 1 Experimental procedures, chiroptical and spectral data of compounds **2**, **2a**, **2b**, **4**, **6**, **8a**, **9a** and **9b**.
