# Supplementary material for: Unconventional application of the Mitsunobu reaction: Selective flavonolignan dehydration yielding hydnocarpins
Source: Beilstein J Org Chem. 2016 Apr 8;12:662–9. doi: 10.3762/bjoc.12.66 (PMC4901995; doi:10.3762/bjoc.12.66)
Supplement: File 1 — Experimental procedures, chiroptical and spectral data of compounds 2, 2a, 2b, 4, 6, 8a, 9a and 9b. [file Beilstein_J_Org_Chem-12-662-s001.pdf]

Supporting Information

for

Unconventional application of the Mitsunobu reaction: Selective flavonolignan dehydration yielding hydnocarpins

Guozheng Huang[1,2], Simon Schramm[1], Jörg Heilmann[3], David Biedermann[4], Vladimír Křen[4] and Michael Decker*,[1]

Address: [1]Pharmazeutische und Medizinische Chemie, Institut für Pharmazie und Lebensmittelchemie, Julius-Maximilians-Universität Würzburg, Am Hubland, D-97074 Würzburg, Germany, [2]College of Life and Environmental Sciences, Shanghai Normal University, Shanghai, P. R. China, [3]Lehrstuhl für Pharmazeutische Biologie, Institut für Pharmazie, Universität Regensburg, Universitätsstraße 31, D-93053 Regensburg, Germany and [4]Centre of Biotransformation and Biocatalysis, Institute of Microbiology, Czech Academy of Sciences, Videnska 1083, Prague 4, CZ-14220, Czech Republic

Email: Michael Decker - michael.decker@uni-wuerzburg.de

* Corresponding author

Experimental procedures, chiroptical and spectral data of compounds 2, 2a, 2b, 4, 6, 8a, 9a and 9b

Common reagents and solvents were obtained from commercial suppliers. Silibinin (mixture of silybin A and silybin B) was purchased from Sigma-Aldrich. Isosilybin A contains ca 5% isosilybin B. Silychristin A contains ca 5% silychristin B. Before reaction, all flavonolignans were dissolved in dry THF and evaporated in vacuo to remove crystalline water. Tetrahydrofuran (THF) was freshly distilled from sodium/benzophenone under argon atmosphere. Reactions were conducted using dried flasks under nitrogen. Reaction progress was monitored using thin layer chromatography (TLC) on precoated

silica gel GF_{254} plates (Macherey-Nagel GmbH & Co. KG, Düren, Germany) and spots were detected by UV light (254 nm). NMR spectra were recorded with Bruker AV-400 or AV-600 NMR instruments in DMSO-d_6, $CDCl_3$ or CD_3OD. Chemical shifts are expressed in ppm relative to the deuterated solvents applied. Optical rotations were measured with a Rudolph Autopol polarimeter (Rudolph Res. Anal., Hackettstown, NJ, USA) in EtOH at 22 °C and CD spectra were recorded in a Jasco-815 spectrometer (Jasco, Easton, MD, USA) in EtOH from 200 to 400 nm with scanning speed 20 nm/min, time response 8 s using 2 mm quartz cell and sample concentration about 1 mmol/L. Analytical HPLC was performed on a Shimadzu LC20AB system equipped with a DGU-20A3R controller, and a SPD-20A UV/Vis detector. Stationary phase was a Synergi 4U fusion-RP (150 × 4.6 mm) column. Gradient MeOH/water (phase A/ phase B) were used as mobile phase. Gradient Mode 1 (GM1): 0–20 min (10–60% phase A), 20–35 min (60% phase A), 35–50 min (60-80% phase A), 50–55 min (80% phase A), 55–65 min (80–10% phase A), 65–70 min (10% phase A). Gradient Mode 2 (GM2): 0–23 min (50–80% phase A), 23–25 min (80% phase A), 25–29 min (80–50% phase A), 29–30 min (50% phase A). ESIMS spectral data were acquired on a Shimadzu LCMS-2020 single quadrupole LC–MS (Shimadzu Europe, Duisburg, Germany).

Synthesis of 23-*O*-[2,2-dimethyl-3-(nitrooxy)]propionylsilibinin (8a) and 2,3-dehydro-23-*O*-[2,2-dimethyl-3-(nitrooxy)]propionylsilibinin (9a)

To the solution of silibinin (145 mg, 0.3 mmol) in dried THF (15 mL) 2,2-dimethyl-3-(nitrooxy)propanoic acid (98 mg, 0.6 mmol, 2.0 equiv) and triphenylphosphine (197 mg, 0.75 mmol, 2.5 equiv) were added. To the mixture a solution of diisopropyl azodicarboxylate (147 µL, 152 mg, 0.76 mmol, 2.5 equiv) in dried THF (7 mL) was added dropwise in cooled water bath (~10 °C) within 3 h. The addition rate was kept slow so that no solid precipitated in the reaction mixture. After addition, the mixture was stirred at room temperature for 1 h. The reaction was quenched with water, and extracted with ethyl acetate (3 × 30 mL). The combined organic phase was washed with brine, dried over anhydrous Na_2SO_4 and solvent evaporated. The residue was purified by column chromatography (CH_2Cl_2/MeOH/NH_4OH 10/1/0.2) to give the products **8a** and **9a**.

23-*O*-[2,2-Dimethyl-3-(nitrooxy)]propionylsilibinin (8a)

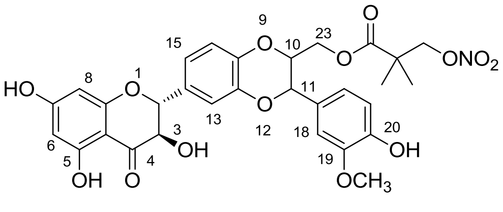

Chemical Formula: $C_{30}H_{29}NO_{14}$
Exact Mass: 627.1588
Molecular Weight: 627.5496

Yellow foam (73 mg, 39 %). **ESI-MS**: 626.1 [M - H]$^+$. **^{1}H NMR** (400 MHz, $CDCl_3$) δ 11.200 (s, 1H, 5-OH), 7.164/7.157 (d, *J* = 1.6 Hz, 1H, 13-H), 7.074/7.060 (dd, *J* = 8.1, 1.8 Hz, 1H, 15-H), 7.013/7.004 (d, *J* = 8.3 Hz, 1H, 16-H), 6.945 (d, *J* = 8.5 Hz, 1H, 21-H), 6.894 – 6.840 (m, 2H, 18-H, 22-H), 6.00 (d, *J* = 1.3 Hz, 1H, 6-H), 5.931/5.924 (dd, *J* = 2.2 Hz, 1H, 8-H), 4.965 (d, *J* = 11.9 Hz, 1H, 2-H), 4.808/4.803 (dd, *J* = 8.1 Hz, 1H, 11-H), 4.545 – 4.456 (m, 3H, 3-H & CH_2ONO_2), 4.356 – 4.284 (m, 1H, 23a-H), 4.265 – 4.188 (m, 1H, 10-H),

4.042/4.036 (dd, J = 12.1, 4.5 Hz, 1H, 23b-H), 3.902 (s, 3H, 19-OCH_3), 1.288/1.283 [s, 6H, $(CH_3)_2$]. **^{13}C NMR** (101 MHz, $CDCl_3$) δ 195.76 (4-CO), 174.34 (1'-COO), 165.89 (7-C), 163.85 (5-C), 163.19 (8a-C), 147.25 (19-C), 146.77 (20-C), 143.98 (16a-C), 143.78 (12a-C), 129.53 (14-C), 127.40 (17-C), 121.50/121.32 (15-C), 120.90 (22-CH), 117.59/117.49 (16-CH), 116.71/116.56 (13-CH), 114.98 (21-CH), 109.47 (18-CH), 100.86 (4a-C), 97.24 (6-CH), 96.12 (8-CH), 83.08/83.04 (2-CH), 77.49 (CH_2ONO_2), 76.71/76.65 (11-CH), 75.80/75.77 (10-CH), 72.43/72.40 (3-CH), 63.57 (23-CH_2O), 56.19 (19-OCH_3), 42.46 (CH_3CCH_3), 22.56 (CH_3CCH_3), 22.45 (CH_3CCH_3).

23-*O*-[2,2-Dimethyl-3-(nitrooxy)]propionylhydnocarpin D (9a)

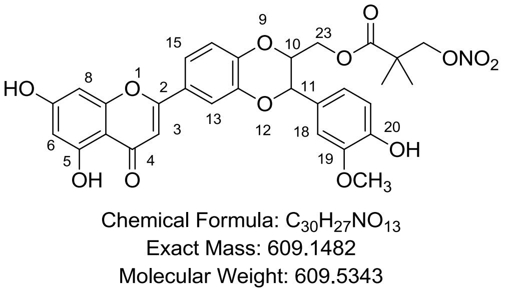

Yellow foam (11 mg, 6%). **ESI-MS**: 608.1 [M - H]$^+$. **^{1}H NMR** (400 MHz, $CDCl_3$) δ 12.78 (s, 1H, 5-OH), 7.52 (d, J = 2.2 Hz, 1H, 13-H), 7.45 (dd, J = 8.6, 2.2 Hz, 1H, 15-H), 7.07 (d, J = 8.6 Hz, 1H, 16-H), 6.99 (d, J = 8.0 Hz, 1H, 22-H), 6.94 – 6.87 (m, 2H, 18-H & 21-H), 6.56 (s, 1H, 3-H), 6.41 (d, J = 2.2 Hz, 1H, 8-H), 6.29 (d, J = 2.2 Hz, 1H, 6-H), 5.76 (s, 1H, 20-OH), 4.87 (d, J = 8.1 Hz, 1H, 11-H), 4.53/4.49 (d, J = 10.3 Hz, 2H, CH_2ONO_2), 4.40 – 4.30 (m, 2H, 10-H & 23a-H), 4.12 – 4.03(m, 1H, 23b-H), 3.94 (s, 3H, 19-OCH_3), 1.30 (s, 6H, CH_3CCH_3). **^{1}H NMR** (400 MHz, DMSO-d_6) δ 12.88 (s, 1H, 5-OH), 10.83 (brs, 1H, 7-OH), 9.25 (s, 1H, 20-OH), 7.71 (d, J = 2.2 Hz, 1H, 13-H), 7.65 (dd, J = 8.6, 2.2 Hz, 1H, 15-H), 7.11 (d, J = 8.6 Hz, 1H, 16-H), 7.07 (d, J = 1.9 Hz, 1H, 18-H), 6.90 (dd, J = 8.2, 1.9 Hz, 1H, 22-H), 6.88 (s, 1H, 3-H), 6.82 (d, J = 8.1 Hz, 1H, 21-H), 6.51 (d, J = 2.1 Hz, 1H, 8-H), 6.20 (d, J = 2.1 Hz, 1H, 6-H), 5.04 (d, J = 8.0 Hz, 1H, 11-H), 4.70 – 4.65 (m, 1H, 10-H), 4.63/4.59 (d, J = 10.3 Hz, 2H, CH_2ONO_2), 4.28 (dd, J = 12.5, 2.8 Hz, 1H, 23a-H), 3.96 (dd, J = 12.5, 4.2 Hz, 1H, 23b-H), 3.78 (s, 3H, 19-OCH_3), 1.20 (d, J = 2.0 Hz, 6H, CH_3CCH_3). **^{13}C NMR** (101 MHz, DMSO-d_6) δ 182.48 (4-CO), 174.15 (COO), 164.21 (2-C), 163.60 (7-C), 161.63 (5-C), 157.97 (9-C), 147.79 (19-C), 147.24 (20-C), 146.24 (12a-C), 143.96 (16a-C), 126.51 (17-C), 124.76 (14-C), 120.70 (22-CH), 120.39 (15-CH), 117.72 (16-CH), 115.48 (21-CH), 115.33 (13-CH), 109.83 (18-CH), 104.68 (4a-C), 104.38 (3-CH), 99.35 (6-CH), 94.35 (8-CH), 76.68 (10-CH), 76.06 (11-CH), 63.28 (23-CH_2O), 55.97 (OCH_3), 42.33 (CH_3CCH_3), 22.35 (CH_3), 22.25 (CH_3).

Hydnocarpin D (**2**)

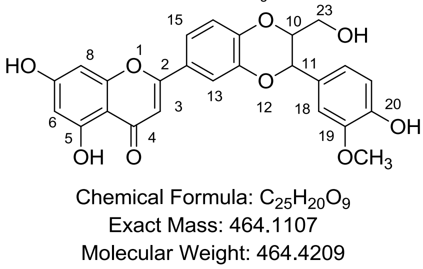

Hydrolysis preparation: To the solution of 23-*O*-[2,2-dimethyl-3-(nitrooxy)]propionylhydnocarpin-D (**7a**, 5.3 mg, 0.0087 mmol) in THF (1 mL) aqueous solution of LiOH (4 M, 1 mL) was added. The mixture was stirred at rt for 2 h until TLC indicated complete deesterification. The reaction mixture was acidified with 1 M HCl to pH < 4, and extracted with ethyl acetate (3 × 10 mL). The combined

organic phase was washed with brine, dried over anhydrous Na_2SO_4 and solvent evaporated. The residue was purified by column chromatography (CH_2Cl_2/MeOH = 20/1) to give title compound **2** as a white solid (3.3 mg, 81%).

One-pot preparation: Silibinin (482 mg, 1 mmol) was dissolved in 100 mL of dried THF. To the solution Ph_3P (1.57 g, 6.0 mmol) and *p*-nitrobenzoic acid (501 mg, 3.0 mmol) was added, then a solution of diisopropyl azodicarboxylate (808 mg, 4.0 mmol) in dried THF (50 mL) at 60 °C was added dropwise. After addition, the mixture was stirred at 60 °C for 1 h, then evaporated in vacuo until 20 mL of THF was left. To this mixture 2 N NaOH solution (20 mL) was added and stirred at rt for 1hrs until TLC indicated complete hydrolysis. The reaction mixture was acidified with 2 M HCl to pH <4, and extracted with CH_2Cl_2 (3 × 30 mL). The combined organic phases were washed with brine, dried over anhydrous Na_2SO_4, and solvent evaporated. The residue was purified by column chromatography (CH_2Cl_2/MeOH/NH_4OH 20/1/0.1) to give title compound **2** as a yellow solid (258 mg, 55.6%). **ESI-MS:** 465.10 (M+H); 463.12 (M -H). **HPLC** purity: 100%, t_R= 9.29 min (GM1), t_R= 17.01 min (GM2). **^{1}H NMR** (400 MHz, DMSO-d_6) δ 12.90 (s, 1H, 5-OH), 10.84 (s, 1H, 7-OH), 9.17 (s, 1H, 20-OH), 7.67 (d, *J* = 2.2 Hz, 1H, 13-H), 7.63 (dd, *J* = 8.5, 2.2 Hz, 1H, 15-H), 7.12 (d, *J* = 8.5 Hz, 1H, 16-H), 7.05 (d, *J* = 1.9 Hz, 1H, 18-H), 6.89 (dd, *J* = 8.2, 1.9 Hz, 1H, 22-H), 6.87 (s, 1H, 3-H), 6.82 (d, *J* = 8.1 Hz, 1H, 21-H), 6.50 (d, *J* = 2.1 Hz, 1H, 8-H), 6.19 (d, *J* = 2.1 Hz, 1H, 6-H), 4.99 (s, 1H, 23-OH), 4.96 (d, *J* = 7.9 Hz, 1H, 11-H), 4.35 – 4.28 (m, 1H, 10-H), 3.79 (s, 3H, 19-OCH_3), 3.61 – 3.53 (m, 23a-H), 3.41 – 3.43 (m, 1H, 23b-H). **^{1}H NMR** (400 MHz, CD_3OD) δ 7.55 (d, *J* = 1.9 Hz, 1H, 13-H), 7.53 (dd, *J* = 8.4, 2.2 Hz, 1H, 15-H), 7.11 (d, *J* = 8.6 Hz, 1H, 16-H), 7.05 (d, *J* = 1.9 Hz, 1H, 18-H), 6.94 (dd, *J* = 8.2, 1.9 Hz, 1H, 22-H), 6.86 (d, *J* = 8.1 Hz, 1H, 21-H), 6.62 (s, 1H, 3-H), 6.45 (d, *J* = 2.1 Hz, 1H, 8-H), 6.21 (d, *J* = 2.1 Hz, 1H, 6-H), 4.97 (d, *J* = 8.1 Hz, 1H, 11-H), 4.17 (ddd, *J* = 8.1, 4.3, 2.5 Hz, 1H, 10-H), 3.89 (s, 3H, 19-OCH_3), 3.75 (dd, *J* = 12.5, 2.5 Hz, 1H, 23a-H), 3.51 (dd, *J* = 12.5, 4.3 Hz, 1H, 23b-H). **^{13}C NMR** (101 MHz, DMSO-d_6) δ 181.76 (4-CO), 164.28 (2-C), 162.91 (7-C), 161.41 (5-C), 157.33 (9-C), 147.66 (19-C), 147.15 (20-C), 146.86 (12a-C), 143.97 (16a-C), 127.03 (17-C), 123.43 (14-C), 120.66 (22-CH), 120.12 (15-CH), 117.35 (16-CH), 115.34 (21-CH), 115.03 (13-CH), 111.79 (18-CH), 103.85 (3-CH), 103.77 (4a-C), 98.91 (6-CH), 94.07 (8-CH), 78.56 (10-CH), 75.91 (11-CH), 60.03 (23-CH_2O), 55.72 (19-OCH_3). NMR data are consistent with that of reference. [1]

(10*R*,11*R*)- hydnocarpin D (2a)

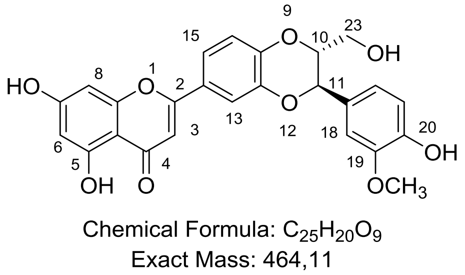

Chemical Formula: $C_{25}H_{20}O_9$
Exact Mass: 464,11
Molecular Weight: 464,43

Silybin A (50 mg, 0.104 mmol, 1.0 eq, purity >95%, were dissolved in THF (20 mL) and PPh_3 (164 mg, 0.624 mmol, 6 equiv) and *p*-nitrobenzoic acid (52 mg, 0.312 mmol, 3 equiv) were added. To this mixture a solution of DIAD (122.5 µL, 0.624 mmol, 6 equiv) in 15 mL of THF were added dropwise (over 1 h) at room temperature. After the addition the mixture was stirred at room temperature overnight. 15 mL of 2 M sodium

hydroxide were added and the mixture was stirred for 1.5 h at room temperature. The mixture was acidified to pH >4 with 2 M HCl and extracted with dichloromethane (3 × 20 mL). The combined organic phases were washed with brine, dried over sodium sulfate and the solvent was removed. The residue was purified by column chromatography (CH_2Cl_2/MeOH 30:1 → 25:1 →20:1) to give title compound **2a** (16 mg, 0.035 mmol, 33%) as a yellow solid. **HPLC** purity: 98.7%, t_R= 17.09 min (GM2). **ESI-MS:** 465.15 $[M+H]^+$. **^{1}H NMR** (400 MHz, acetone-d_6) δ 12.97 (s, 1H, 5-OH), 7.62 (dd, *J* = 9.0, 2.0 Hz, 1H, 15-H), 7.61 (d, *J* = 2.0 Hz, 1H, 13-H), 7.17 (d, *J* = 2.0 Hz, 1H, 18-H), 7.10 (d, *J* = 9.0 Hz, 1H, 16-H), 7.01 (dd, *J* = 8.2, 1.9 Hz, 1H, 22-H), 6.91 (d, *J* = 8.0 Hz, 1H, 21-H), 6.69 (s, 1H, 3-H), 6.59 (d, *J* = 1.5 Hz, 1H, 8-H), 6.26 (d, *J* = 1.5 Hz, 1H, 6-H), 5.06 (d, *J* = 8.0 Hz, 1H, 11-H), 4.28 (ddd, *J* = 8.2, 3.9, 2.8 Hz, 1H, 10-H), 3.89 (s, 3H, 19-OCH_3), 3.80 (dd, *J* = 12.5, 2.5 Hz, 1H, 23a-H), 3.55 (dd, *J* = 12.4, 3.9 Hz, 1H, 23b-H). **^{1}H NMR** (400 MHz, CD_3OD) δ 7.56 (d, *J* = 2.0 Hz, 1 H, 13-H), 7.53 (dd, *J* = 8.3, 2.3 Hz, 1 H, 15-H), 7.13 (d, *J* = 8.8 Hz, 1H, 16-H), 7.05 (d, *J* = 1.8 Hz, 1H, 18-H), 6.94 (dd, *J* = 8.3, 2.0 Hz, 1H, 22-H), 6.86 (d, *J* = 8.0 Hz, 1H, 21-H), 6.63 (s, 1H, 3-H), 6.46 (d, *J* = 2.0 Hz, 1H, 8-H), 6.21 (d, *J* = 1.8 Hz, 1H, 6-H), 4.98 (d, *J* = 8.0 Hz, 1H, 11-H), 4.18 (ddd, *J* = 8.0, 4.4, 2.4 Hz, 1H, 10-H), 3.89 (s, 3H, 19-OCH_3), 3.75 (dd, *J* = 12.3, 2.3 Hz, 1H, 23a-H), 3.51 (dd, *J* = 12.3, 4.3 Hz, 1H, 23b-H). **^{13}C NMR** (101 MHz, acetone-d_6) δ 182.17 (4-CO), 164.22 (2-C), 163.51 (7-C), 162.46 (5-C), 157.92 (9-C), 147.69 (19-C), 147.32 (20-C), 147.26 (12a-C), 144.47 (16a-C), 127.85 (17-C), 124.12 (14-C), 120.85 (22-CH), 119.98 (15-CH), 117.44 (16-CH), 115.05 (21-CH), 114.94 (13-CH), 111.13 (18-CH), 104.49 (3-CH), 103.97 (4a-C), 98.93 (6-CH), 93.97 (8-CH), 79.21 (10-CH), 76.42 (11-CH), 60.80 (23-CH_2O), 55.51 (19-OCH_3).

(*10S,11S*)- Hydnocarpin D (2b)

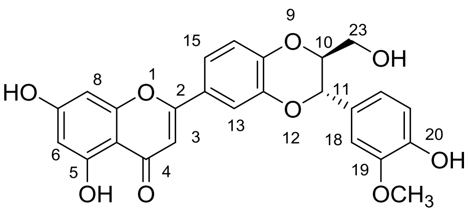

Chemical Formula: $C_{25}H_{20}O_9$
Exact Mass: 464,11
Molecular Weight: 464,43

Silybin B (50 mg, 0.104 mmol, 1.0 equiv, purity>95%,) were dissolved in THF (20 mL) and PPh_3 (164 mg, 0.624 mmol, 6 equiv) and *p*-nitrobenzoic acid (52 mg, 0.312 mmol, 3 equiv) were added. To this mixture a solution of DIAD (122.5 µL, 0.624 mmol, 6 equiv) in 15 mL of THF were added dropwise (over 1 h) at room temperature. After the addition the mixture was stirred at room temperature overnight. 15 mL of 2 M sodium hydroxide were added and the mixture was stirred for 1.5 h at room temperature. The mixture was acidified to pH >4 with 2 M HCl and extracted with dichloromethane (3 × 20 mL). The combined organic phases were washed with brine, dried over sodium sulfate and the solvent was removed. The residue was purified by column chromatography (CH_2Cl_2/MeOH 30:1 →20:1) to give title compound **2b** (18 mg, 0.039 mmol, 37%) as a yellow solid. **HPLC** purity: 97.0%, t_R= 17.05 min (GM2). **ESI-MS:** 465.15 $[M+H]^+$. **^{1}H NMR** (400 MHz, acetone-d_6) δ 12.97 (s, 1H, 5-OH), 7.62 (dd, *J* = 8.8, 2.3 Hz, 1H, 15-H), 7.61 (d, *J* = 2.0 Hz, 1H, 13-H), 7.17 (d, *J* = 1.8 Hz, 1H, 18-H), 7.10 (d, *J* = 9.0 Hz, 1H, 16-H), 7.01 (dd, *J* = 8.0, 2.0 Hz, 1H, 22-H), 6.91 (d, *J* = 8.0 Hz, 1H, 21-H), 6.69 (s, 1H, 3-H), 6.59 (d, *J* = 1.5 Hz, 1 H), 6.26 (d, *J* = 1.5 Hz, 1 H),

5.06 (d, J = 8.0 Hz, 1H, 11-H), 4.28 (ddd, J = 8.3, 4.1, 2.6 Hz, 1H, 10-H), 3.89 (s, 3H, 19-OCH_3), 3.80 (dd, J = 12.5, 2.5 Hz, 1H, 23a-H), 3.55 (dd, J = 12.4, 4.1 Hz, 1H, 23b-H). **^{1}H NMR** (400 MHz, CD_3OD) δ 7.57 (d, J = 2.0 Hz, 1 H, 13-H), 7.55 (dd, J = 8.3, 2.3 Hz, 1 H, 15-H), 7.13 (d, J = 8.3 Hz, 1H, 16-H), 7.05 (d, J = 2.0 Hz, 1H, 18-H), 6.94 (dd, J = 8.3, 1.8 Hz, 1H, 22-H), 6.86 (d, J = 8.0 Hz, 1H, 21-H), 6.63 (s, 1H, 3-H), 6.46 (d, J = 2.3 Hz, 1H, 8-H), 6.21 (d, J = 2.0 Hz, 1H, 6-H), 4.98 (d, J = 8.0 Hz, 1H, 11-H), 4.17 (ddd, J = 8.3, 4.3, 2.5 Hz, 1H, 10-H), 3.89 (s, 3H, 19-OCH_3), 3.75 (dd, J = 12.5, 2.5 Hz, 1H, 23a-H), 3.51 (dd, J = 12.4, 4.1 Hz, 1H, 23b-H).**^{13}C NMR** (101 MHz, acetone-d_6) δ 182.09 (4-CO), 163.98 (2-C), 163.46 (7-C), 162.16 (5-C), 157.92 (9-C), 147.65 (19-C), 147.25 (2C, 20-C, 12a-C), 144.46 (16a-C), 127.85 (17-C), 124.10 (14-C), 120.85 (22-CH), 119.97 (15-CH), 117.44 (16-CH), 115.08 (21-CH), 114.88 (13-CH), 111.14 (18-CH), 104.50 (3-CH), 104.03 (4a-C), 98.79 (6-CH), 93.97 (8-CH), 79.19 (10-CH), 76.41 (11-CH), 60.72 (23-CH_2O), 55.51 (19-OCH_3).

23-*O*-Benzoylhydnocarpin D (9b)

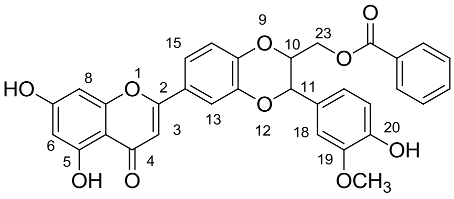

Chemical Formula: $C_{32}H_{24}O_{10}$
Exact Mass: 568.1369
Molecular Weight: 568.5270

To the solution of silibinin (48 mg, 0.1 mmol) in dried THF (8 mL) benzoic acid (36 mg, 0.3 mmol, 2.0 equiv) and PPh_3 (156 mg, 0.6 mmol) were added. To the mixture a solution of diisopropyl azodicarboxylate (100 mg, 0.4 mmol, 2.5 equiv) in dried THF (4 mL) was added dropwise in cold water bath (~10 °C) within 4 hrs. The addition rate was kept slow so that no solid precipitated in the reaction mixture. After the addition, the mixture was stirred at room temperature for 20 h. The reaction was quenched with water, and extracted with ethyl acetate (3 × 30 mL). The combined organic phases were washed with brine, dried over anhydrous Na_2SO_4, and solvent evaporated. The residue was purified by column chromatography (CH_2Cl_2/MeOH/NH_4OH 10/1/0.1) to give the title compound **9b** as a white solid (19 mg, 33.4%). **HPLC** purity: 100%, t_R= 10.31min (GM1). **ESI-MS:** 585.50 (M + Na). **^{1}H NMR** (400 MHz, $CDCl_3$) δ 12.83 (s, 1H, 5-OH), 8.04 – 7.98 (m, 2H, 2 × *ortho*-Ph), 7.59 (d, J = 7.5 Hz, 1H, *para*-Ph), 7.55 (d, J = 2.2 Hz, 1H, 13-H), 7.50 – 7.43 (m, 3H, 15-H, 2 × *meta*-Ph), 7.12 (d, J = 8.6 Hz, 1H, 16-H), 6.97 – 6.94 (m, 2H, 18-H, 22-H), 6.88 (s, 1H, 21-H), 6.57 (s, 1H, 3-H), 6.41 (d, J = 2.2 Hz, 1H, 8-H), 6.28 (d, J = 2.2 Hz, 1H, 6-H), 5.68 (s, 1H, OH), 5.38 (s, 1H, OH), 5.05 (d, J = 8.1 Hz, 1H, 11-H), 4.58 (dd, J = 12.4, 3.0 Hz, 1H, 23b-H), 4.49 – 4.44 (m, 1H, 10-H), 4.29 (dd, J = 12.4, 4.2 Hz, 1H, 23b-H), 3.83 (s, 3H, 19-OCH_3). **^{1}H NMR** (400 MHz, CD_3OD) δ 7.96 (d, J = 7.2 Hz, 2H, 2 × *ortho*-Ph), 7.61 (t, J = 7.4 Hz, 1H, *para*-Ph), 7.57 (d, J = 2.1 Hz, 1H, 13-H), 7.53 (dd, J = 8.6, 2.1 Hz, 1H, 15-H), 7.48 (t, J = 7.8 Hz, 2H, 2 × *meta*-Ph), 7.11 (d, J = 8.5 Hz, 1H, 16-H), 7.02 (d, J = 1.6 Hz, 1H, 18-H), 6.94 (dd, J = 8.1, 1.7 Hz, 1H, 22-H)), 6.84 (d, J = 8.1 Hz, 1H, 21-H), 6.61 (s, 1H, 3-H), 6.44 (d, J = 2.1 Hz, 1H, 8-H), 6.21 (d, J = 2.0 Hz, 1H, 6-H), 5.07 (d, J = 7.9 Hz, 1H, 11-H), 4.57 (ddd, J = 7.6, 3.8, 3.4 Hz, 1H, 10-H), 4.52 (dd, J = 12.2, 3.4 Hz, 23a-H), 4.29 (dd, J = 12.2, 3.9 Hz, 1H, 23b-H), 3.77 (s, 3H, 19-OCH_3). **^{1}H NMR** (400 MHz, DMSO-d_6) δ 12.88 (s, 1H, 5-OH), 10.87 (s, 1H, 7-OH), 9.24 (s, 1H, 20-OH), 7.96 (dd, J = 8.3, 1.2 Hz, 2H, 2 × *ortho*-Ph), 7.72 (d, J = 2.2 Hz, 1H,

13-H), 7.70 – 7.62 (m, 2H, 15-H, , *para*-Ph), 7.54 (t, J = 7.7 Hz, 2H, 2 × *meta*-Ph), 7.18 (d, J = 8.6 Hz, 1H, 16-H), 7.07 (d, J = 1.8 Hz, 1H, 18-H), 6.92 (dd, J = 8.2, 1.8 Hz, 1H, 22-H), 6.88 (s, 1H, 3-H), 6.81 (d, J = 8.1 Hz, 1H, 21-H), 6.50 (d, J = 2.0 Hz, 1H, 8-H), 6.18 (d, J = 2.0 Hz, 1H, 6-H), 5.21 (d, J = 8.0 Hz, 1H, 11-H), 4.85 – 4.76 (m, 1H, 10-H), 4.47 (dd, J = 12.5, 2.8 Hz, 1H, 23a-H), 4.21 (dd, J = 12.5, 4.3 Hz, 1H, 23a-H), 3.68 (s, 3H, 19-OCH_3). **^{13}C NMR** (101 MHz, DMSO-d_6) δ 181.72 (4-CO), 165.33 (2-C), 162.73 (7-C), 161.42 (5-C), 157.39 (9-C), 147.79 (19-C), 147.46 (20-C), 146.33 (12a-C), 144.08 (16a-C), 133.65 (*para*-Ph) , 129.37 (2 × *meta*-Ph), 129.04 (*ipso*-Ph), 128.85 (2 × *para*-Ph), 126.19 (17-C), 123.97 (14-C), 120.75 (22-CH), 120.30 (15-CH), 117.58 (16-CH), 115.51 (21-CH), 115.26 (13-CH), 111.87 (18-CH), 104.04 (3-CH), 103.67 (4a-C), 99.05 (6-CH), 94.19 (8-CH), 76.22 (10-CH), 75.49 (11-CH), 63.25 (23-CH_2O), 55.57 (19-OCH_3). **^{13}C NMR** (151 MHz, DMSO-d_6) δ 181.79 (4-CO), 165.33 (2-C), 162.75 (7-C), 161.42 (5-C), 157.37 (9-C), 147.78 (19-C), 147.45 (20-C), 146.35 (12a-C), 144.10 (16a-C), 133.67 (*para*-Ph) , 129.39 (2 × *meta*-Ph), 129.03 (*ipso*-Ph), 128.86 (2 × *para*-Ph), 126.17 (17-C), 123.94 (14-C), 120.74 (22-CH), 120.31 (15-CH), 117.58 (16-CH), 115.49 (21-CH), 115.29 (13-CH), 111.80 (18-CH), 104.05 (3-CH), 103.74 (4a-C), 98.99 (6-CH), 94.16 (8-CH), 76.19 (10-CH), 75.48 (11-CH), 63.24 (23-CH_2O), 55.53 (19-OCH_3).

Hydnocarpin (4)

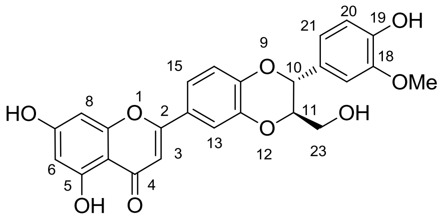

Chemical Formula: $C_{25}H_{20}O_9$
Exact Mass: 464.1107
Molecular Weight: 464.4209

Isosilybin A (19.3 mg, 0.04 mmol, prepared from silymarin based on enzymatic kinetic resolution,[3] containing ca 5% isosilybin B as impurity,) was dissolved in dried THF and concentrated in vacuo to remove water, then dissolved in 2 mL of dry THF. To the solution were added Ph_3P (63 mg, 0.24 mmol, 6.0 equiv) and *p*-nitrobenzoic acid (20 mg, 0.12 mmol, 3.0 equiv), followed by dropwise addition of a solution of diisopropyl azodicarboxylate (32 mg, 0.16 mmol, 4 equiv) in dry THF (2 mL) at room temperature. After addition, the mixture was stirred at r.t for 20 h. To this mixture a solution of 2 N NaOH (2 mL) was added and stirred 1 h at rt until TLC indicated complete hydrolysis. The reaction mixture was acidified with 2 M HCl until pH <4, and extracted with CH_2Cl_2 (3 × 10 mL). The combined organic phases were washed with brine, dried over anhydrous Na_2SO_4, and solvent evaporated. The residue was purified by column chromatography (CH_2Cl_2/MeOH/NH_4OH 10/1/0.1) to give title compound as a yellow solid (**4**, 4.5 mg, 24% yield). **HPLC** purity: 100%, t_R= 8.56 min (GM1). **MS-ESI:** 465.10 [M+H]. ^{1}H NMR (600 MHz, DMSO-d_6) δ 12.92 (s, 1H, 5-OH), 10.88 (s, 1H, 7-OH), 9.20 (s, 1H, 20-OH), 7.68 (d, J = 2.2 Hz, 1H, 13-H), 7.61 (dd, J = 8.5, 2.2 Hz, 1H, 15-H), 7.09 (d, J = 8.5 Hz, 1H, 16-H), 7.04 (d, J = 1.9 Hz, 1H, 18-H), 6.90 (s, 1H, 3-H), 6.88 (dd, J = 8.2, 1.9 Hz, 1H, 22-H), 6.81 (d, J = 8.1 Hz, 1H, 21-H), 6.53 (d, J = 2.1 Hz, 1H, 8-H), 6.20 (d, J = 2.1 Hz, 1H, 6-H), 5.02 (d, J = 7.9 Hz, 1H, 11-H), 5.00 (t, J = 5.2 Hz, 1H, 23-OH), 4.31 – 4.25 (m, 1H, 10-H), 3.78 (s, 3H, 19-OCH_3), 3.62 – 3.55 (m, 1H, 23a-H), 3.41 – 3.36 (m, 1H, 23b-H). **^{13}C NMR** (151 MHz, DMSO-d_6) δ 181.82 (4-CO), 164.31 (2-C), 162.93 (7-C), 161.44 (5-C), 157.36 (9-C), 147.66 (19-C), 147.16 (20-C),

147.13 (12a-C), 143.69 (16a-C), 126.96 (17-C), 123.71 (14-C), 120.63 (22-CH), 119.94 (15-CH), 117.56 (16-CH), 115.34 (21-CH), 114.86 (13-CH), 111.76 (18-CH), 103.95 (3-CH), 103.80 (4a-C), 98.94 (6-CH), 94.13 (8-CH), 78.00 (10-CH), 76.40 (11-CH), 60.08 (23-CH_2O), 55.71 (19-OCH_3). NMR data are in accordance with literature. [2]

Isohydnocarpin (6), number 9 was omitted in the numbering of the structure shown below to facilitate comparison with the hydnocarpin skeleton

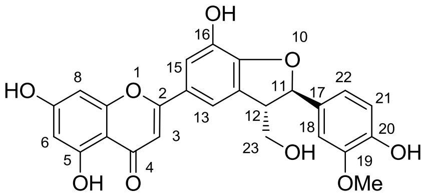

Chemical Formula: $C_{25}H_{20}O_9$
Exact Mass: 464.1107
Molecular Weight: 464.4209

Silychristin A (containing ca 5% of silychristin B as impurity, total 19.3 mg, 0.04 mmol,) was dissolved in dried THF and concentrated in vacuo to remove residual water, then dissolved in 2 mL of dried THF. To the solution Ph_3P (63 mg, 0.24 mmol, 6.0 equiv) and *p*-nitrobenzoic acid (20 mg, 0.12 mmol, 3.0 equiv) were added, followed by drop wise addition of a solution of diisopropyl azodicarboxylate (32 mg, 0.16 mmol, 4 equiv) in dried THF (2 mL) at room temperature. After addition, the mixture was stirred room temperature for 20 h. To this mixture a solution of 2 N NaOH (2 mL) was added and stirred 1 h at rt until TLC indicated complete hydrolysis. The reaction mixture was acidified with 2 M HCl to pH <4, and extracted with CH_2Cl_2 (3 × 10 mL). The combined organic phases were washed with brine, dried over anhydrous Na_2SO_4, and solvent evaporated. The residue was purified by column chromatography (CH_2Cl_2/MeOH/NH_4OH 10/2/0.1) to give 4.1 mg of a yellow solid (22% yield). **HPLC** purity: 100%, t_R= 8.56 min (GM1). **MS-ESI:** 465.10 [M+H]. **^{1}H NMR** (600 MHz, DMSO-d_6) δ 12.96 (s, 1H, 5-OH), 10.89 (s, 1H, 7-OH), 9.77 (s, 1H), 9.09 (s, 1H, 20-OH), 7.51 (dd, J = 1.7, 0.9 Hz, 1H, 15-H), 7.37 (d, J = 1.8 Hz, 1H, 13-H), 6.96 (d, J = 1.9 Hz, 1H, 18-H), 6.81 (dd, J = 8.3, 1.9 Hz, 1H, 22-H), 6.77 (d, J = 8.1 Hz, 1H, 21-H), 6.72 (s, 1H, 3-H), 6.46 (dd, J = 5.2, 2.1 Hz, 1H, 8-H), 6.20 (d, J = 2.1 Hz, 1H, 6-H), 5.59 (d, J = 6.7 Hz, 1H, 11-H), 5.10 (t, J = 5.4 Hz, 1H, 23-OH), 4.55 (brs, 1H, 23-OH), 3.80 – 3.69 (m, 1H, 23a-H), 3.75 (s, 3H, 19-OCH_3), 3.74 – 3.69 (m, 1H, 23b-H), 3.55 (dd, J = 12.1, 6.1 Hz, 1H, 12-H). **^{13}C NMR** (151 MHz, DMSO-d_6) δ 181.69 (4-CO), 164.20 (2-C), 163.90 (8a-C), 161.49 (7-C), 157.32 (5-C), 150.59 (17-C), 147.62 (19-C), 146.61 (16-C), 141.53 (16a-C), 131.85 (17-C), 130.64 (12a-C), 123.43 (14-C), 118.84 (22-CH), 115.39 (13-CH), 114.69 (21-CH), 114.19 (15-CH), 110.49 (18-CH), 103.74 (4a-C), 103.26 (3-CH), 98.88 (6-CH), 93.89 (8-CH), 87.99 (11-CH), 62.59 (23-CH_2O), 55.67 (19-OCH_3), 52.83 (12-CH). [2]

References:

1 N. R. Guz and F. R. Stermitz, *J. Nat. Prod.*, 2000, **63**, 1140.

2 X.-J. Hu, H.-Z. Jin, X.-H. Liu and W.-D. Zhang, *Helv. Chim. Acta*, 2011, **94**, 306.

3 R. Gažák, K. Fuksová, P. Marhol, M. Kuzma, R. Agarwal and V. Křen, *Proc. Biochemistry,* 2013, **48**, 184

<Chromatogram>

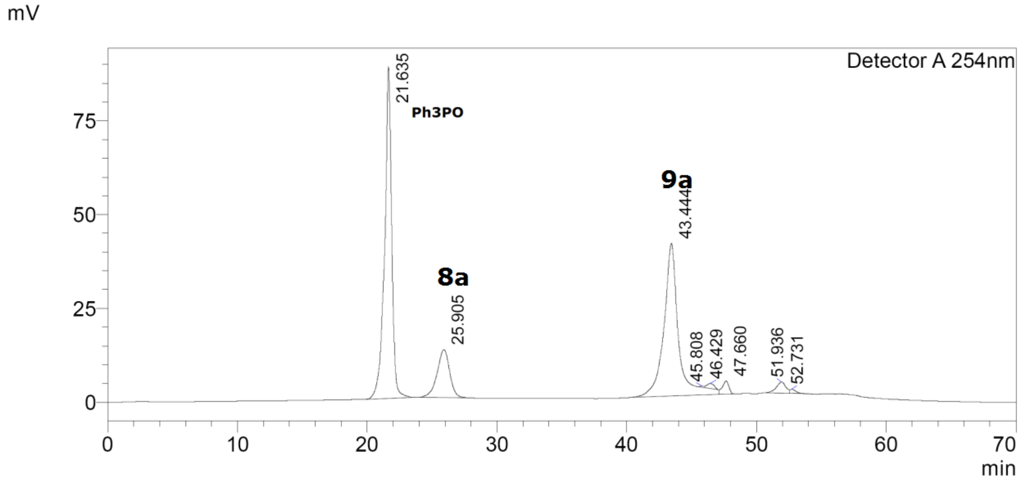

Peak Table

Detector A 254nm

Peak#	Ret. Time	Area	Area%
1	21.635	3354526	43.323
2	25.905	867684	11.206
3	43.444	3149238	40.671
4	45.808	7029	0.091
5	46.429	51944	0.671
6	47.660	126094	1.628
7	51.936	150755	1.947
8	52.731	35868	0.463
Total		7743137	100.000

Fig. S1. Ratio of **8a** and **9a** by HPLC (Entry 3, Table 1 of the main text)

<Chromatogram>

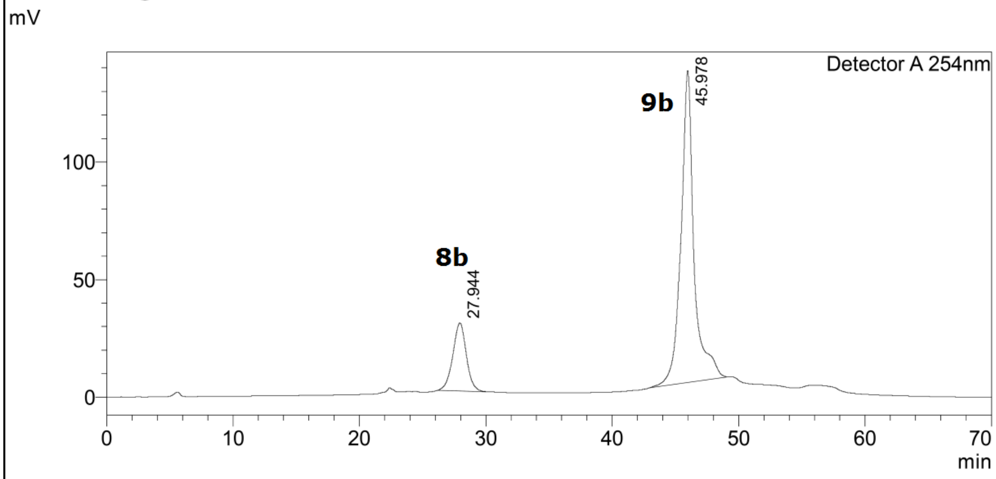

Peak Table

Detector A 254nm

Peak#	Ret. Time	Area	Area%
1	27.944	2155168	18.475
2	45.978	9510224	81.525
Total		11665392	100.000

Fig. S2. Ratio of **8b** and **9b** by HPLC (Entry 3, Table 1 of the main text)

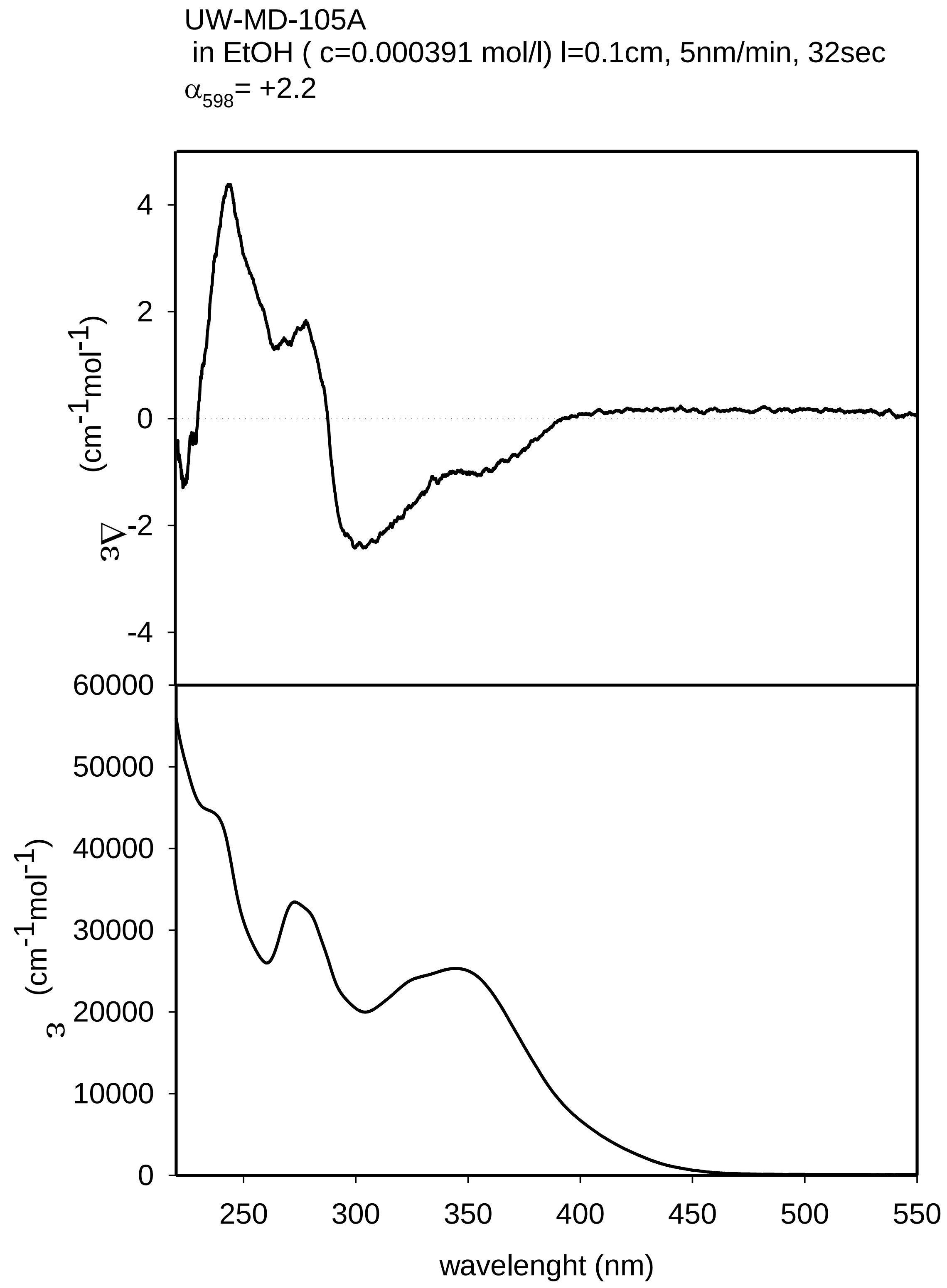

Fig. S3. ECD (upper pannel) and UV (bottom pannel) spectra of compound **2a**; OR $[\alpha]^{22}_{589}$ +2.2 (*c* 0.0039, EtOH).

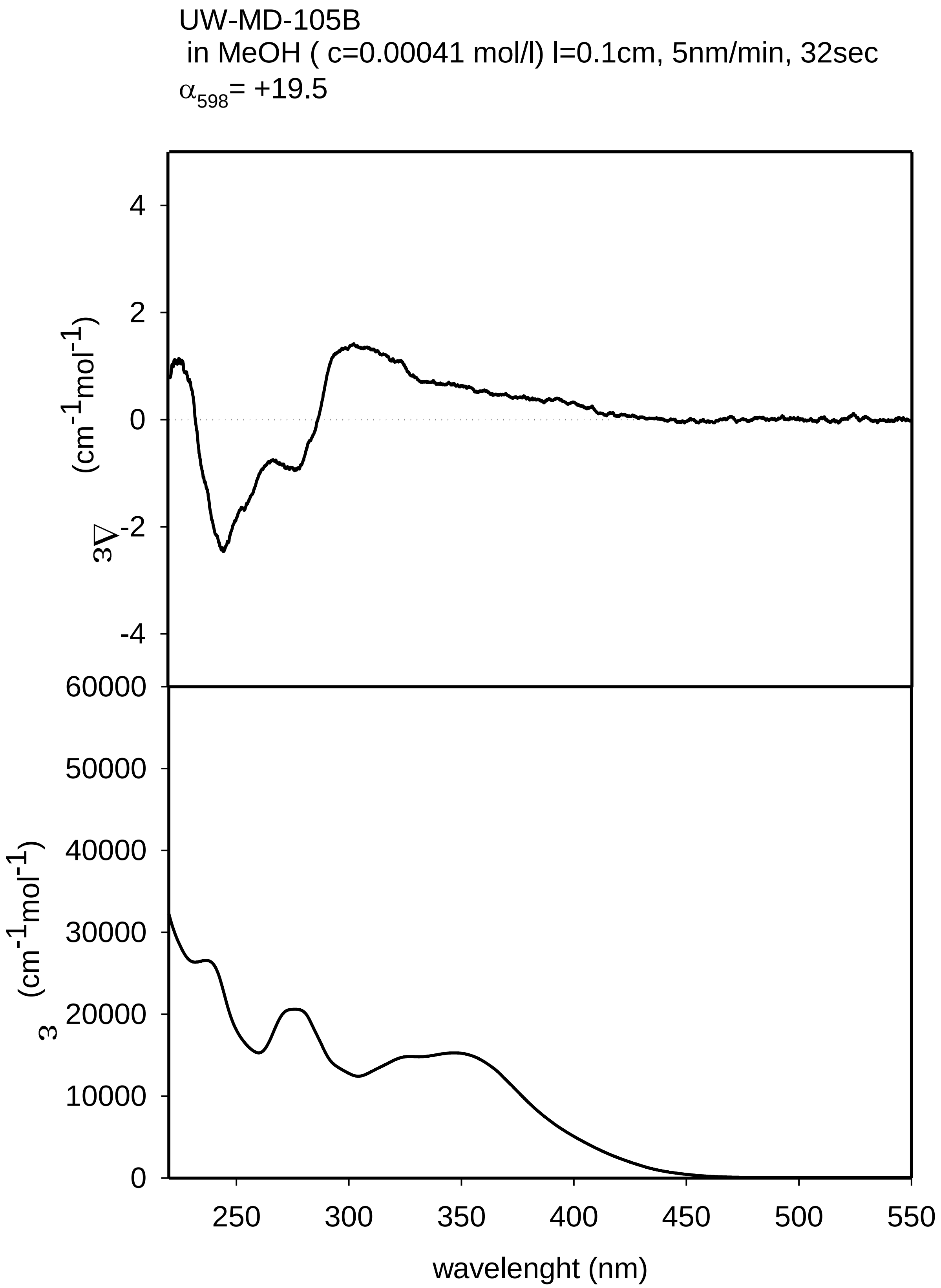

Fig. S4. ECD (upper pannel) and UV (bottom pannel) spectra of compound **2b**; OR $[\alpha]^{22}_{589}$ +19.5 (*c* 0.0041, EtOH).

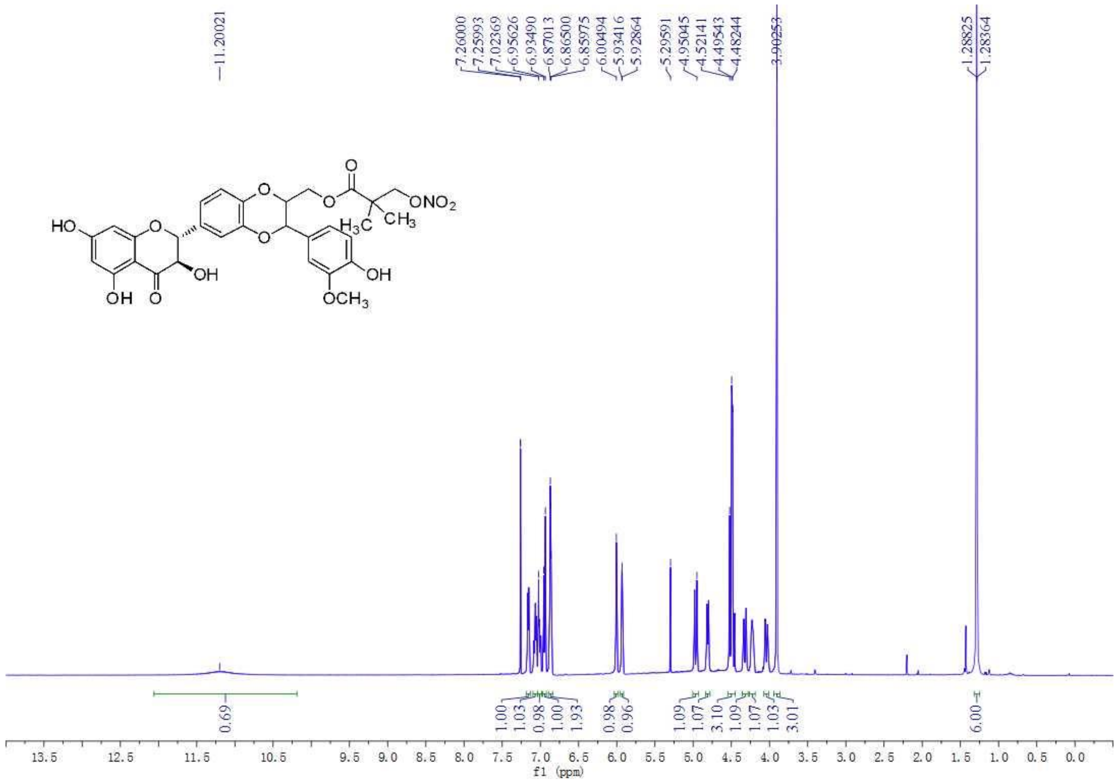

^{1}H NMR (400 MHz, $CDCl_3$) of **8a**

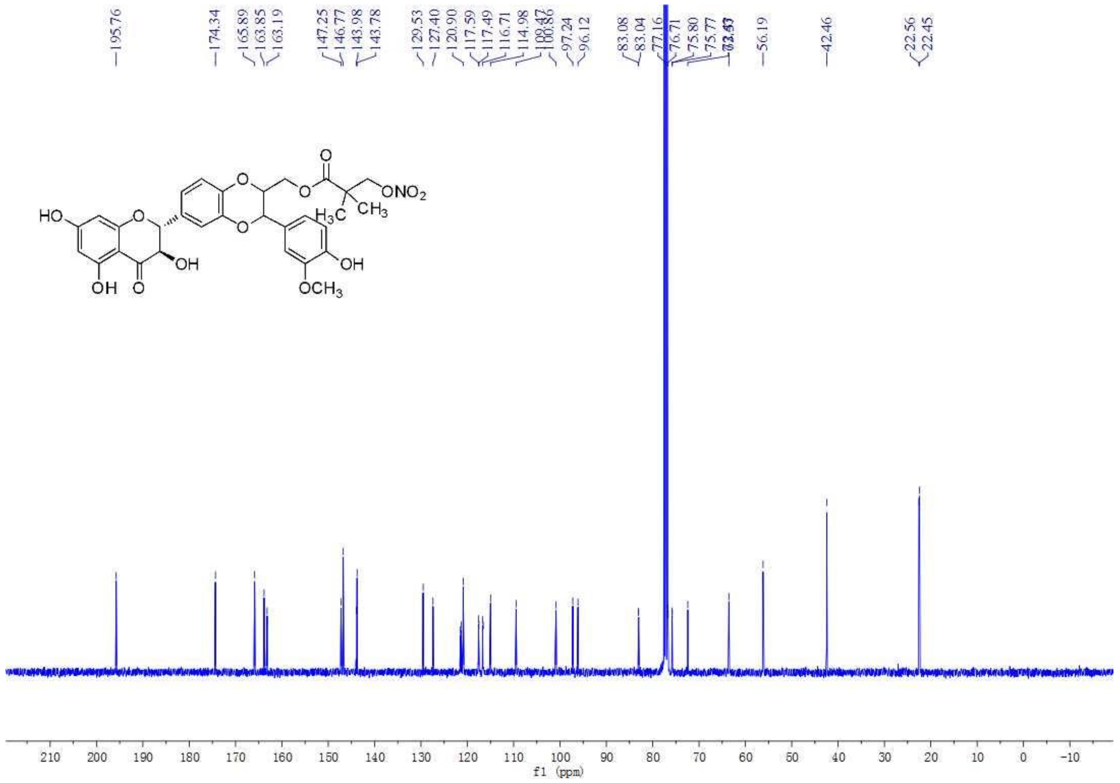

^{13}C NMR (101 MHz, $CDCl_3$) of **8a**

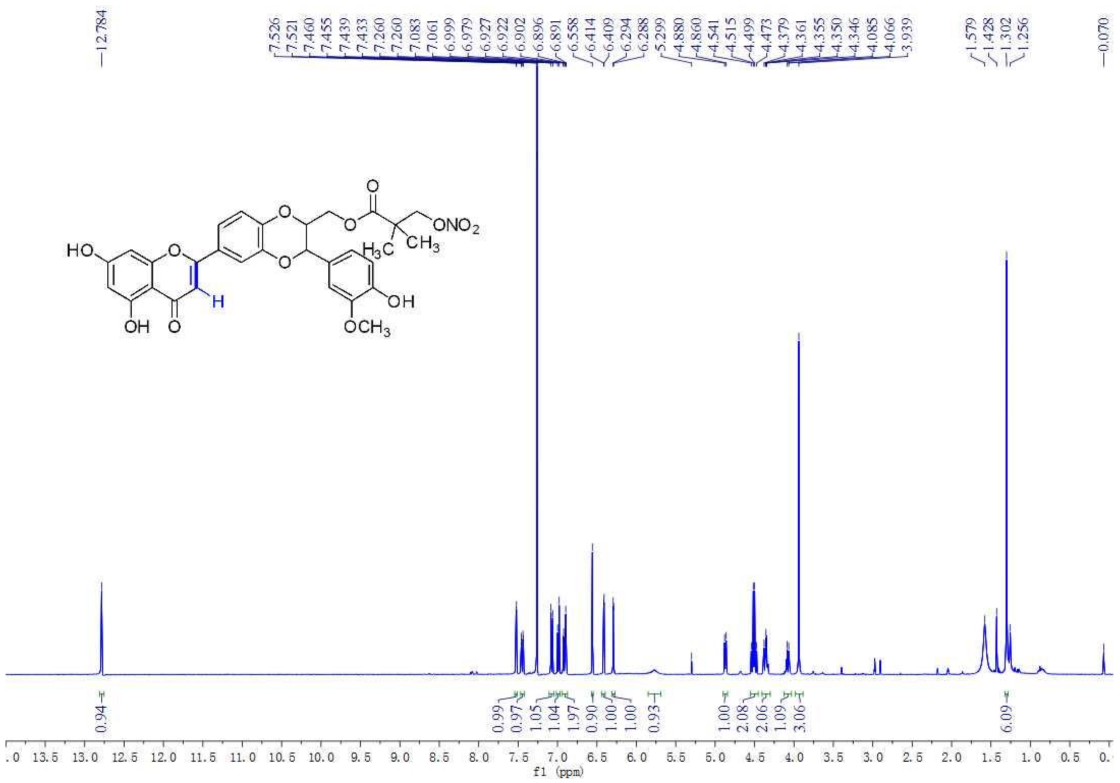

^{1}H NMR (400 MHz, $CDCl_3$) of **9a**

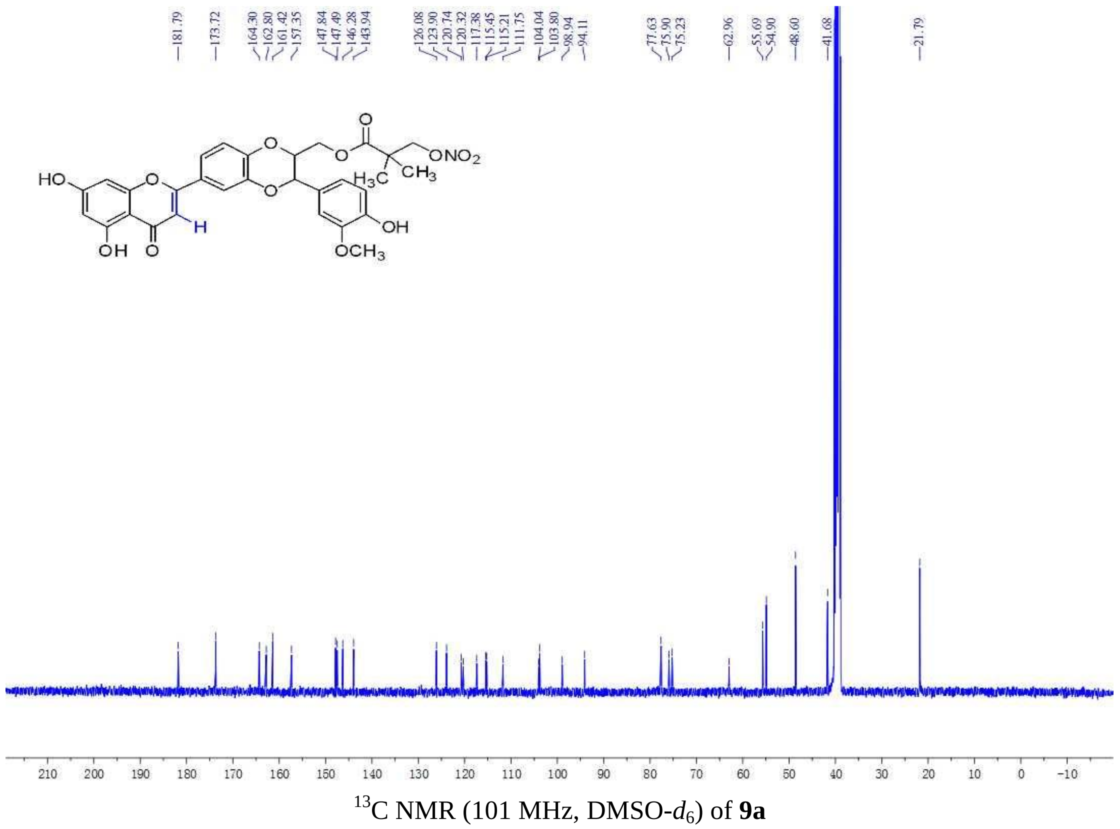

^{13}C NMR (101 MHz, DMSO-d_6) of **9a**

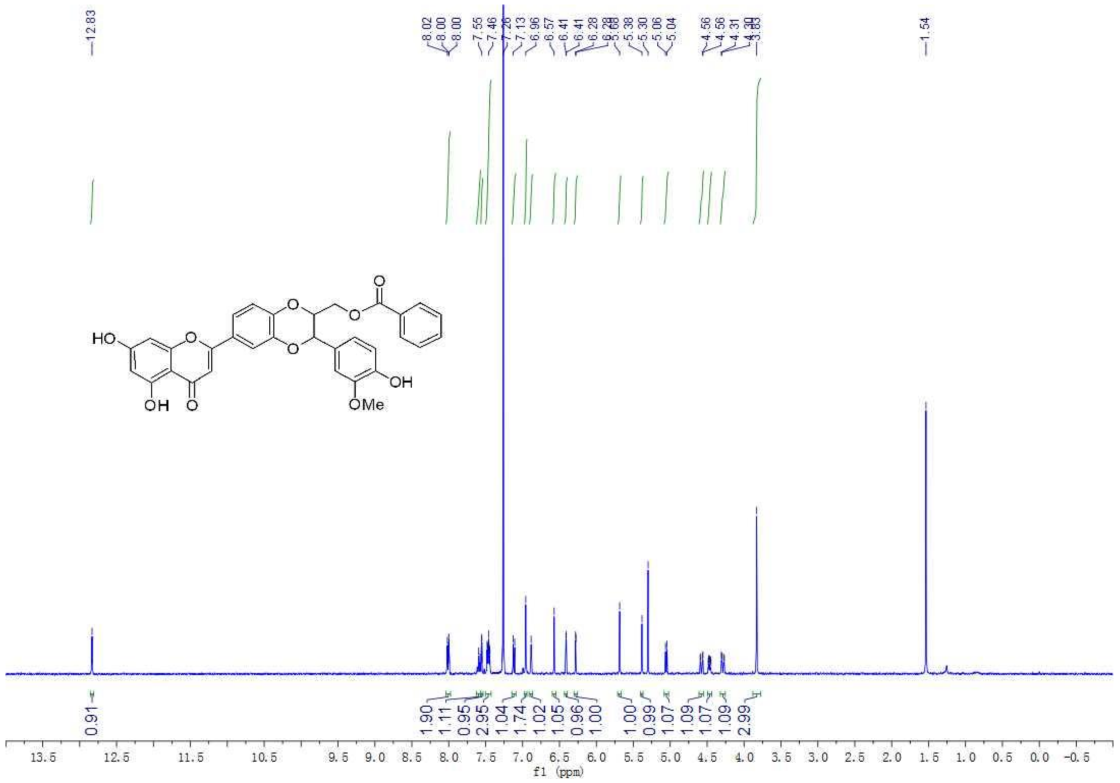

^{1}H NMR (400 MHz, $CDCl_3$) of **9b**

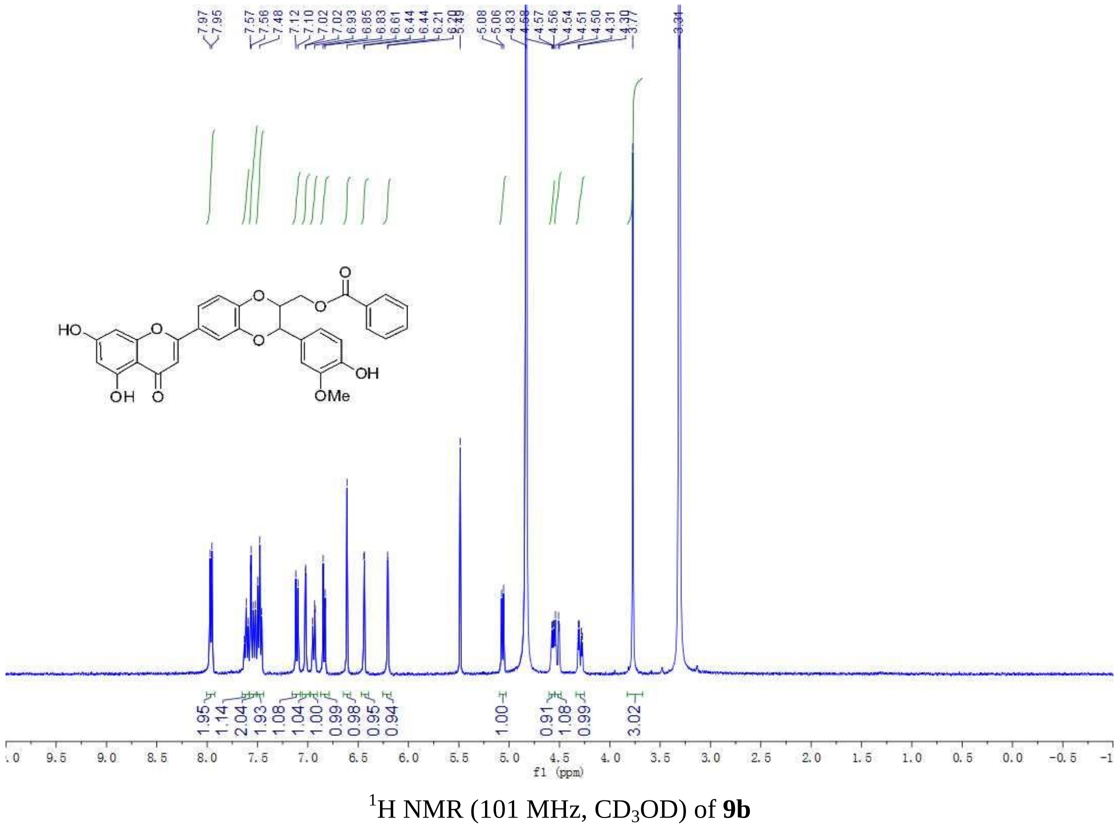

^{1}H NMR (101 MHz, CD_3OD) of **9b**

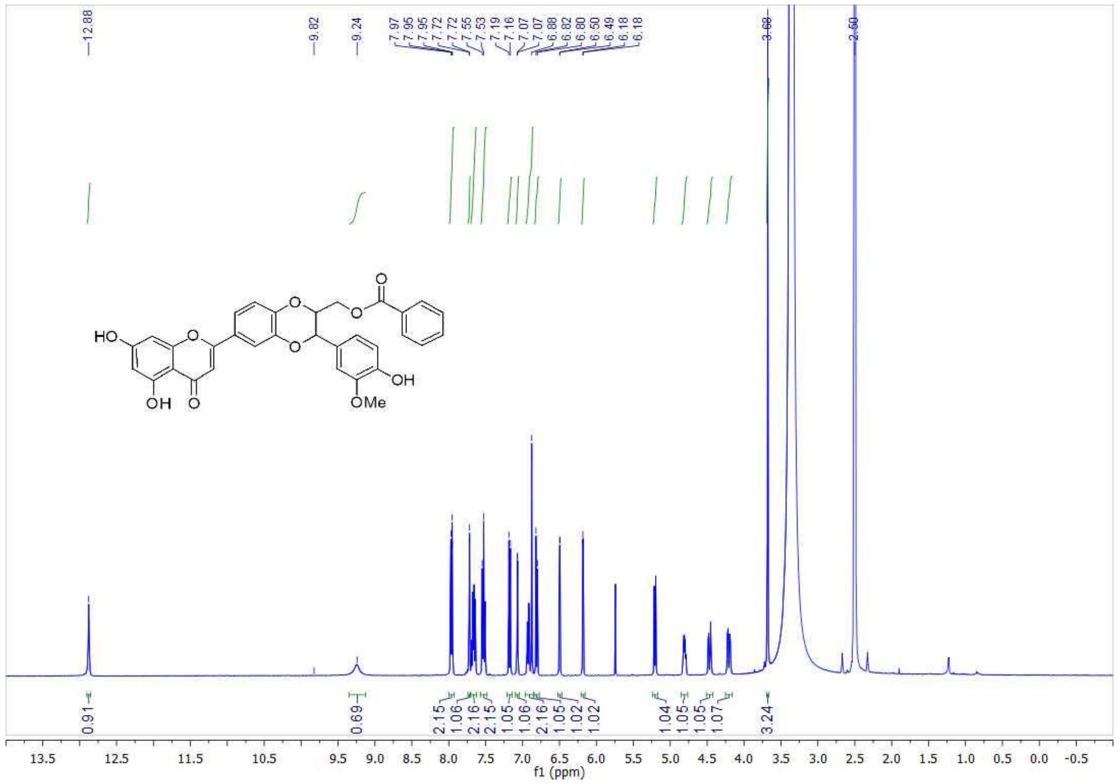

^{1}H NMR (600 MHz, DMSO-d_6) of **9b**

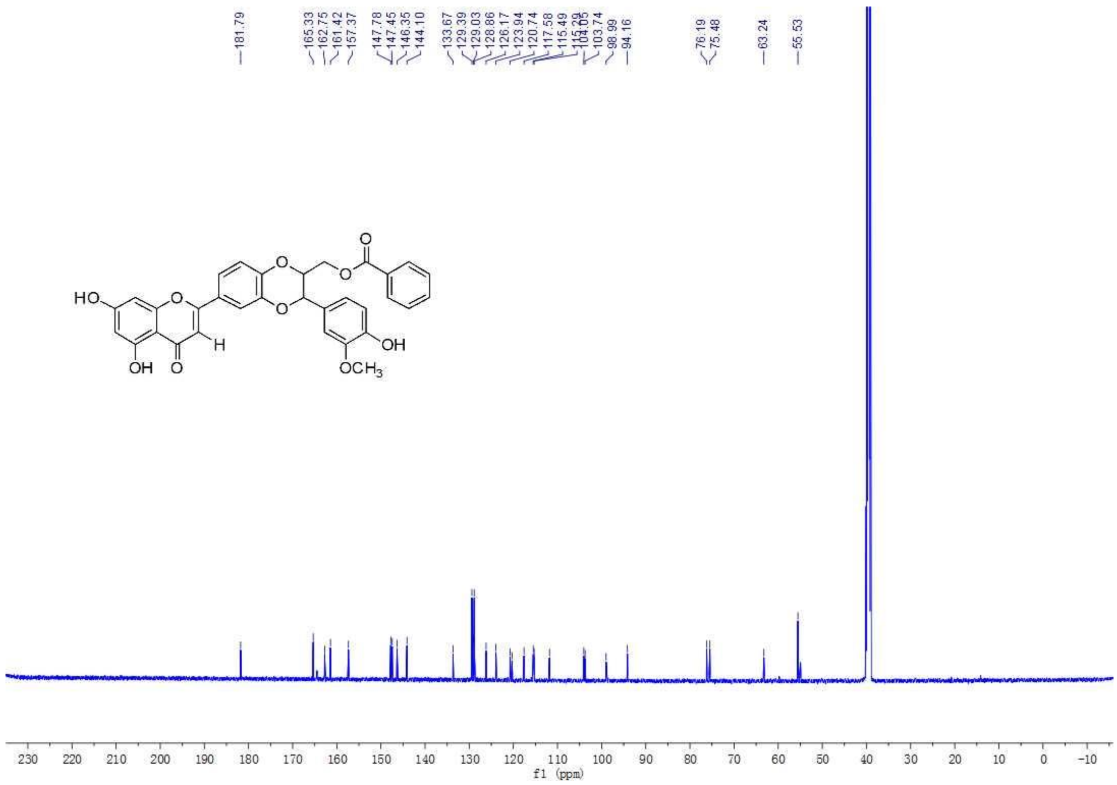

^{13}C NMR (151 MHz, DMSO-d_6) of **9b**

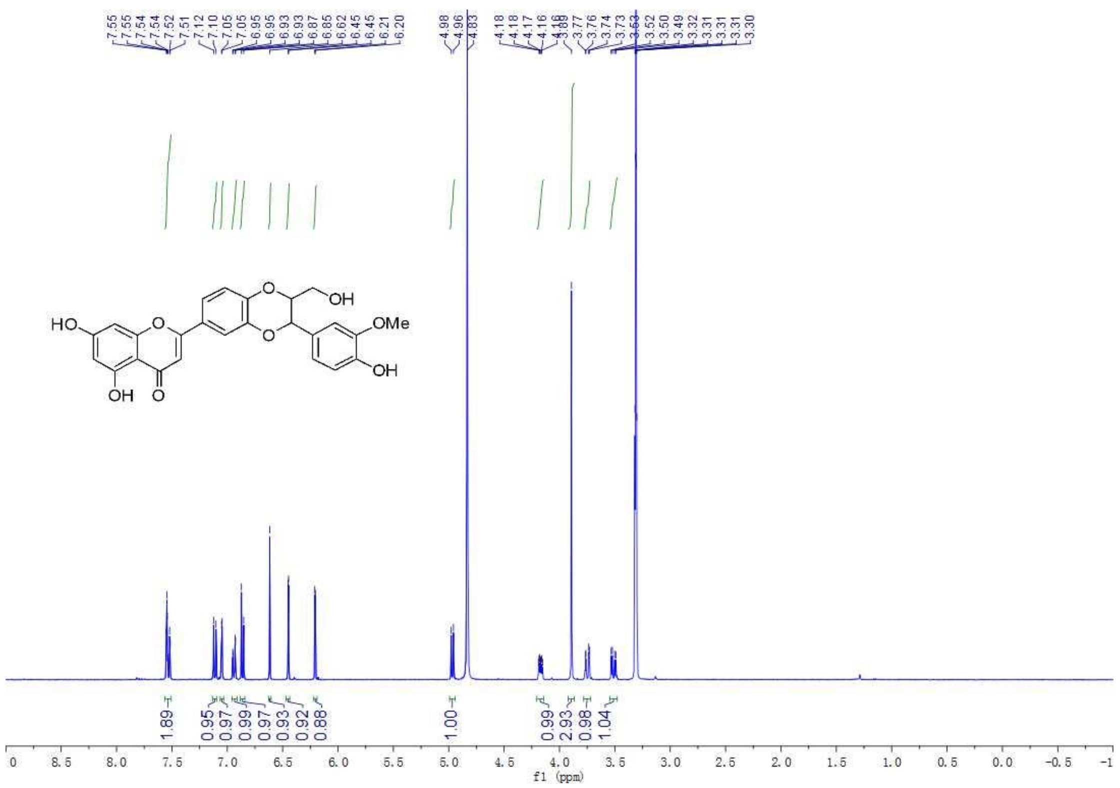

^{1}H NMR (400 MHz, CD_3OD) of **2**

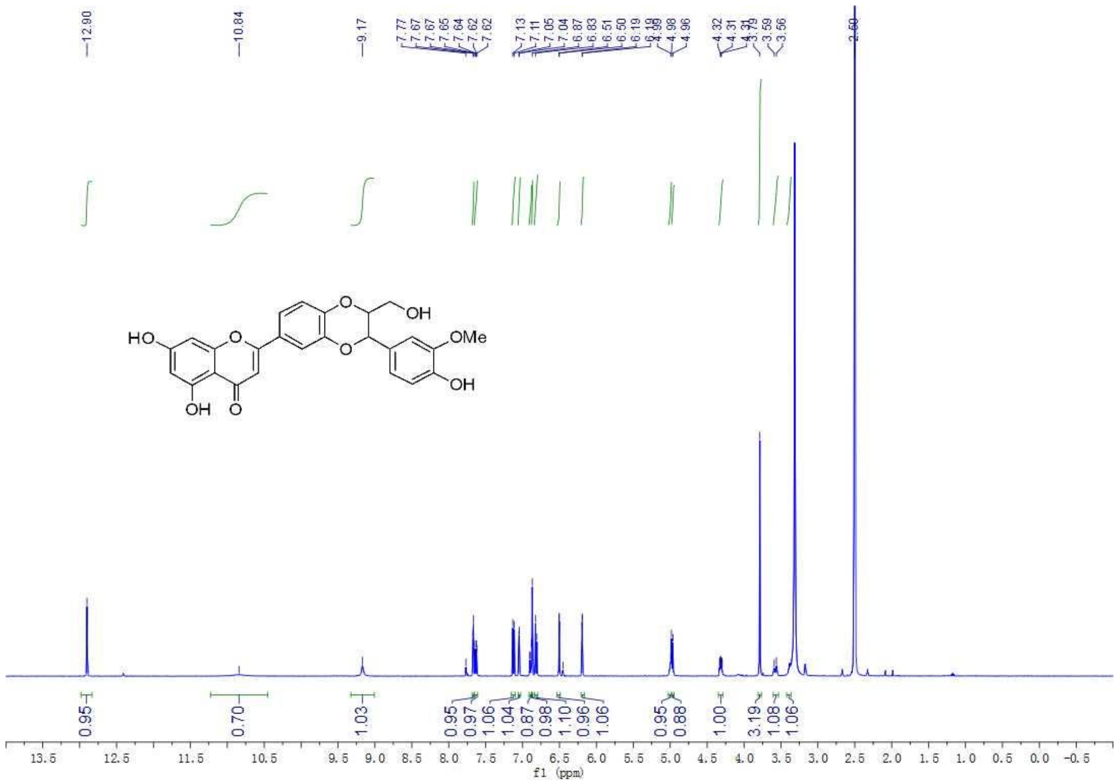

^{1}H NMR (400 MHz, DMSO-d_6) of **2**

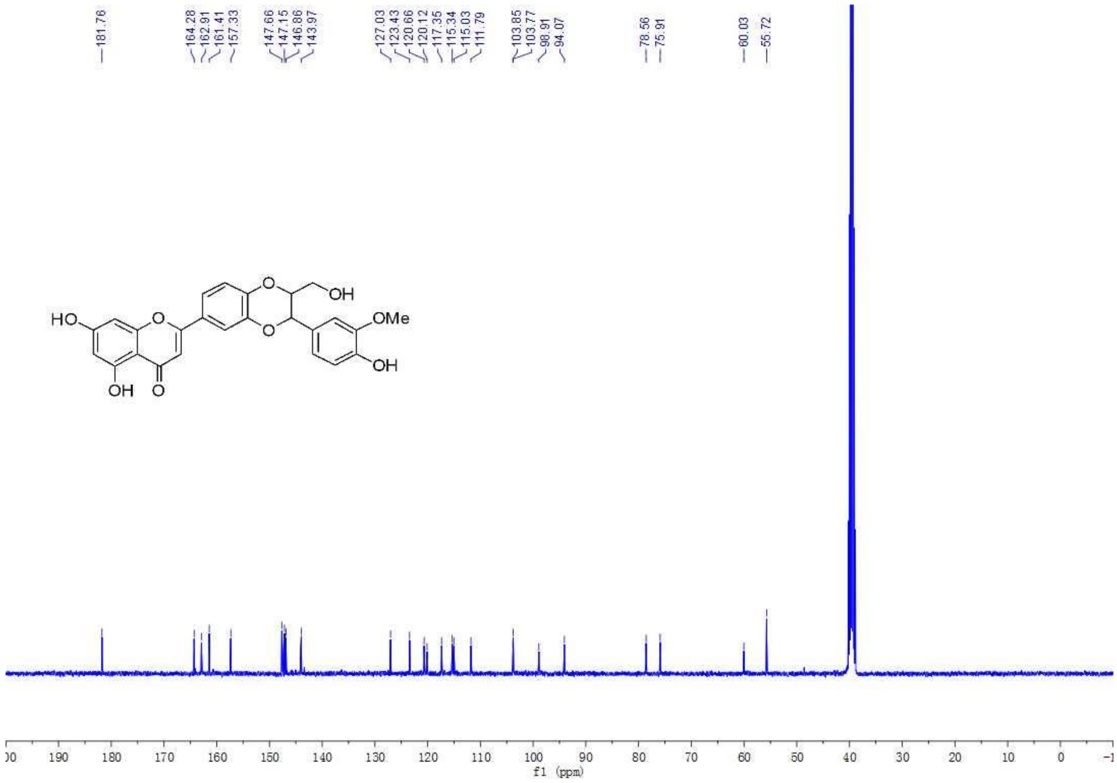

^{13}C NMR (101 MHz, DMSO-d_6) of **2**

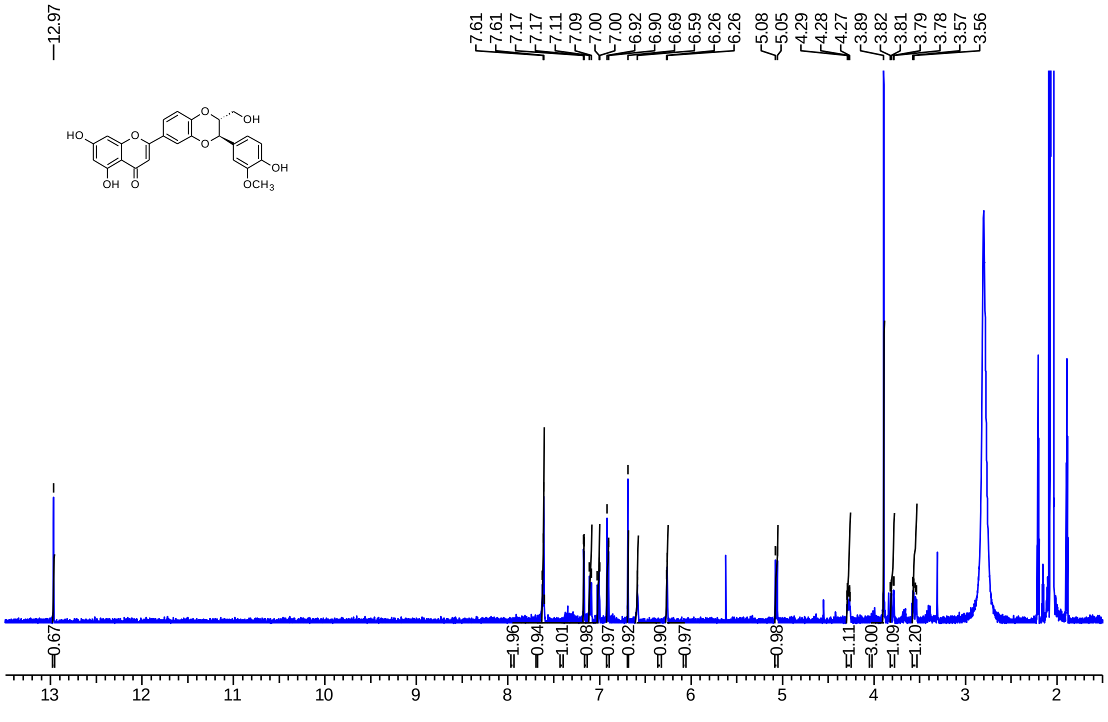

^{1}H NMR (400 MHz, Acetone-d_6) of **2a**

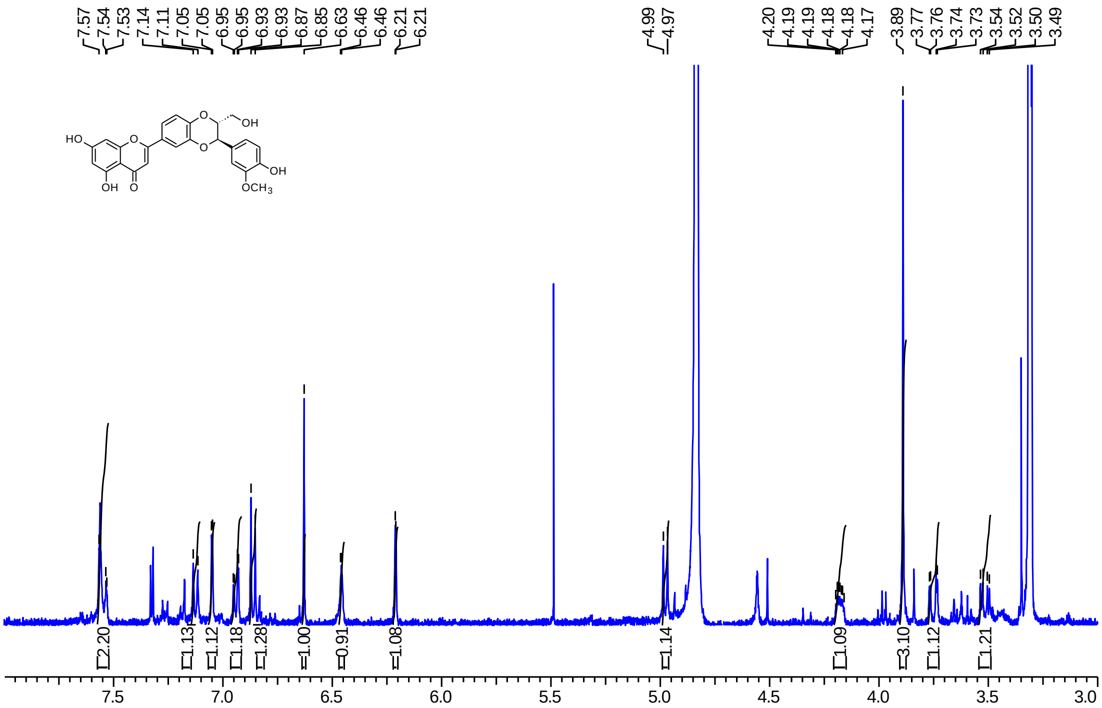

^{1}H NMR (400 MHz, CD_3OD) of **2a**

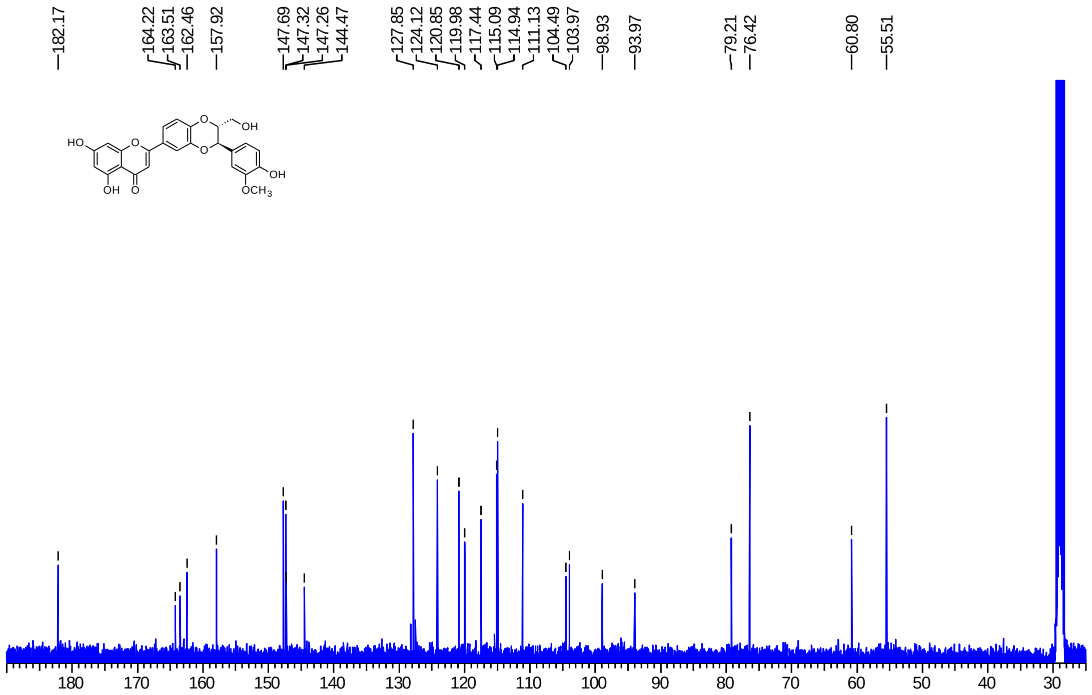

^{13}C NMR (101 MHz, Acetone-d_6) of **2a**

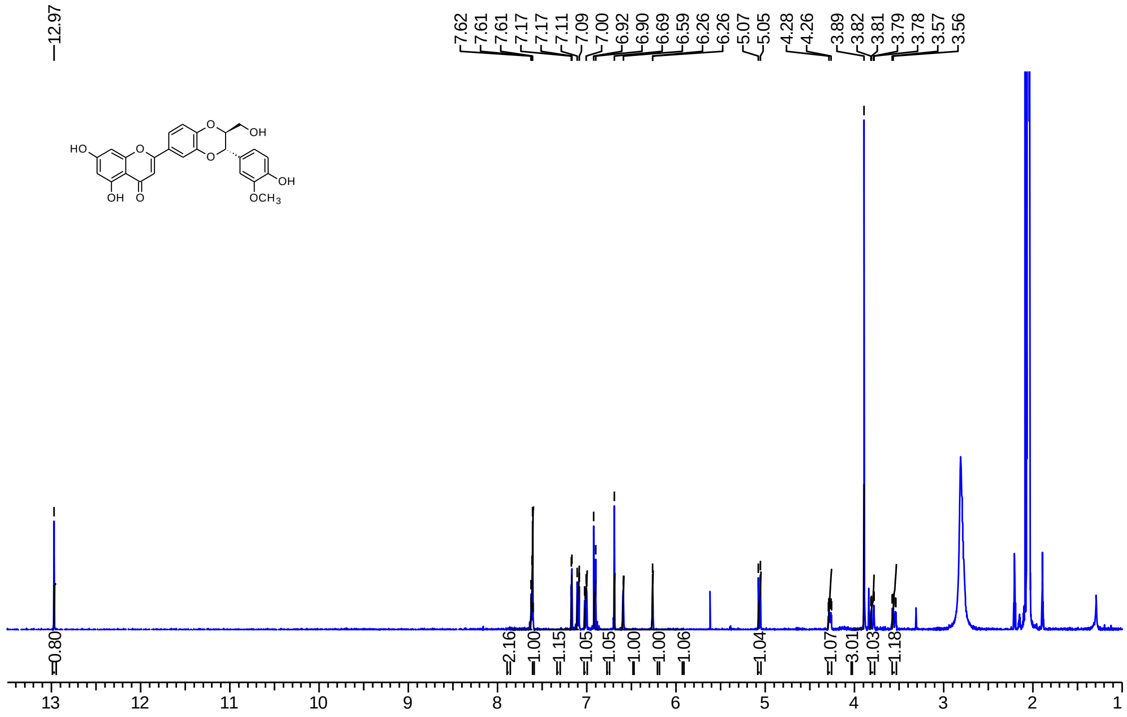

^{1}H NMR (400 MHz, Acetone-d_6) of **2b**

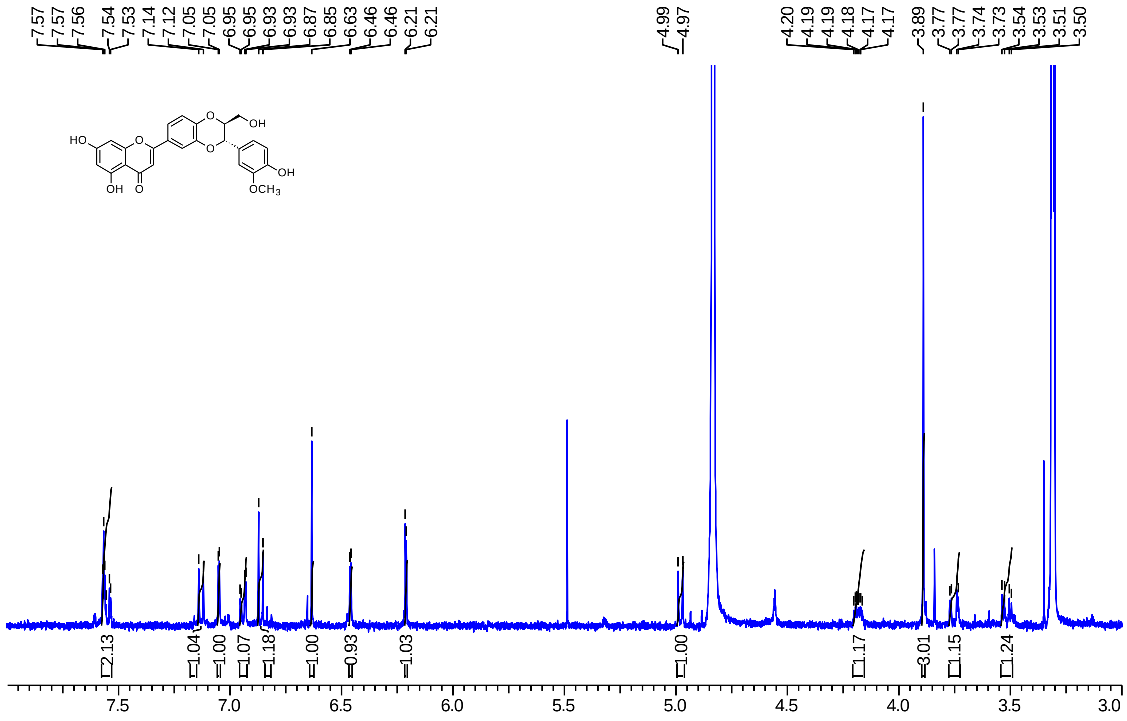

^{1}H NMR (400 MHz, CD_3OD) of **2b**

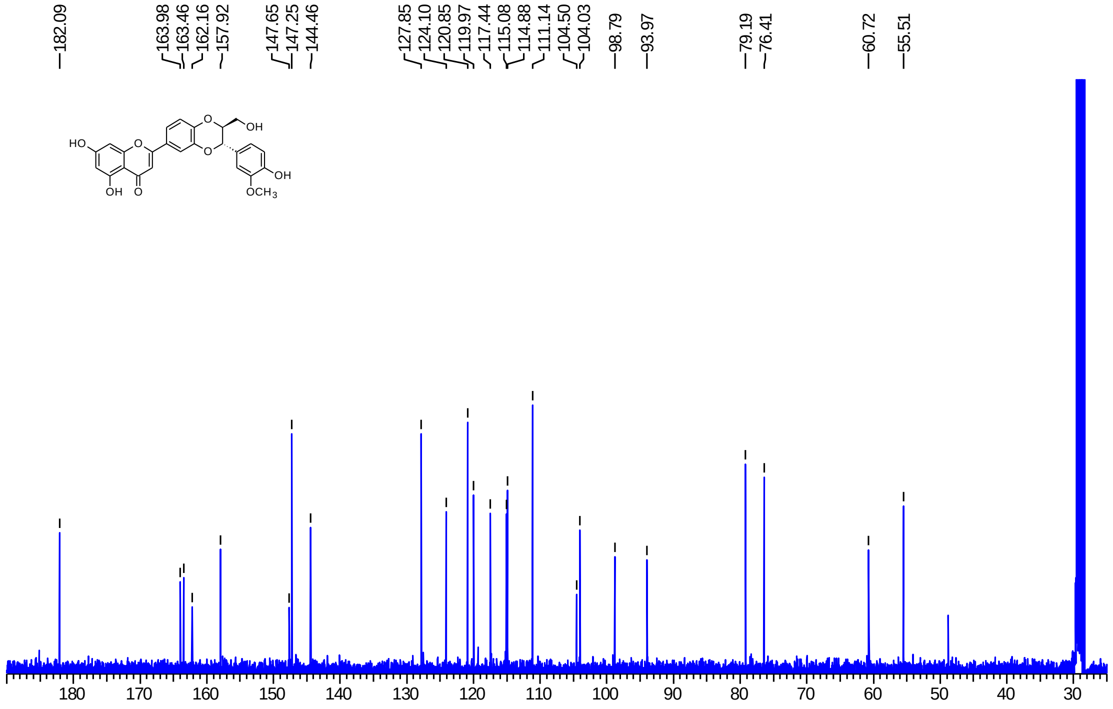

^{13}C NMR (101 MHz, Acetone-d_6) of **2b**

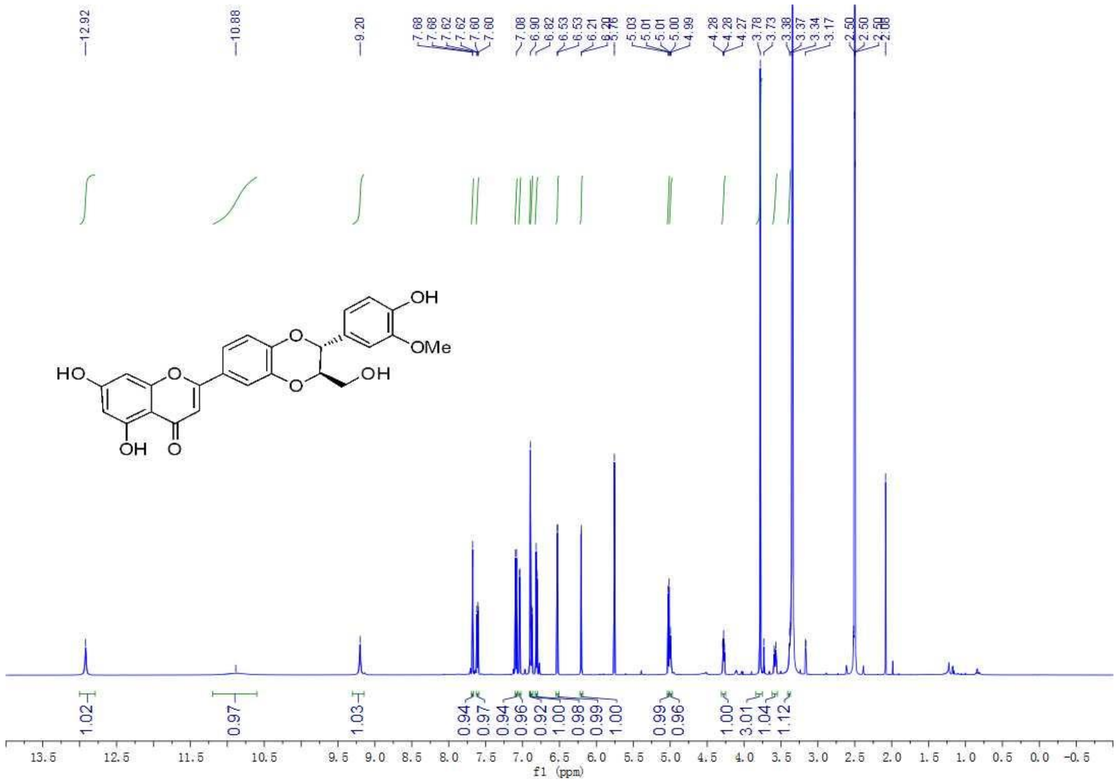

^{1}H NMR (600 MHz, DMSO-d_6) of **4**

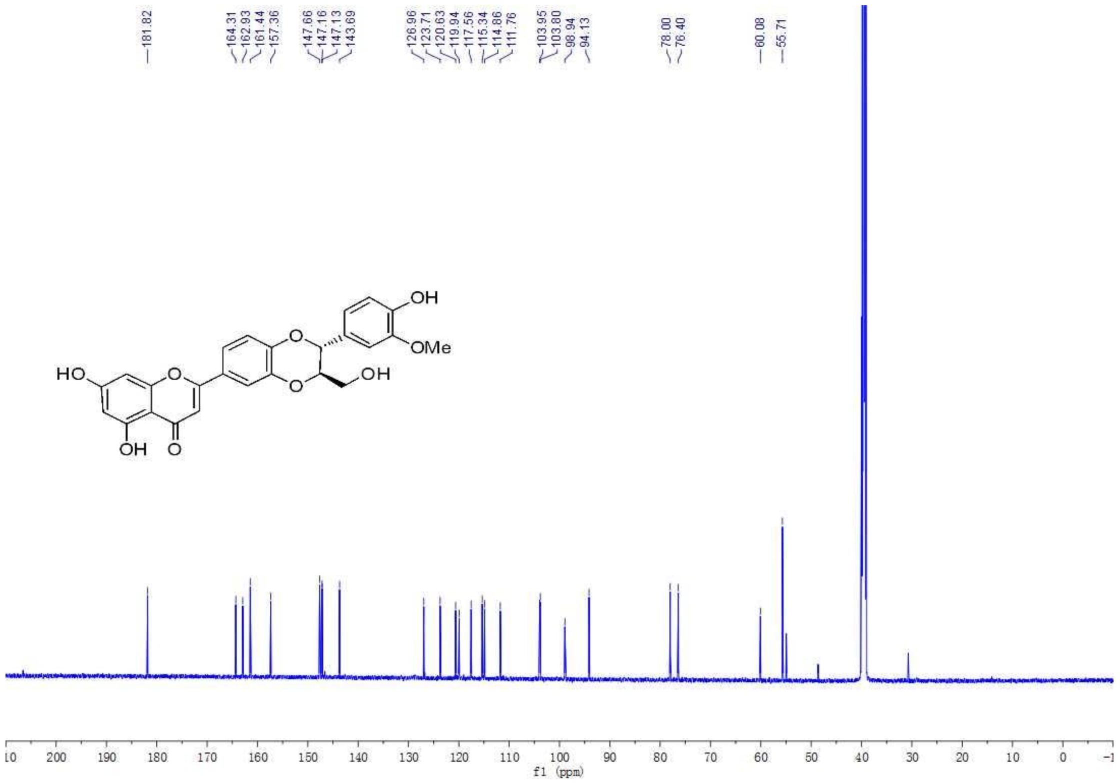

^{13}C NMR (151MHz, DMSO-d_6) of **4**

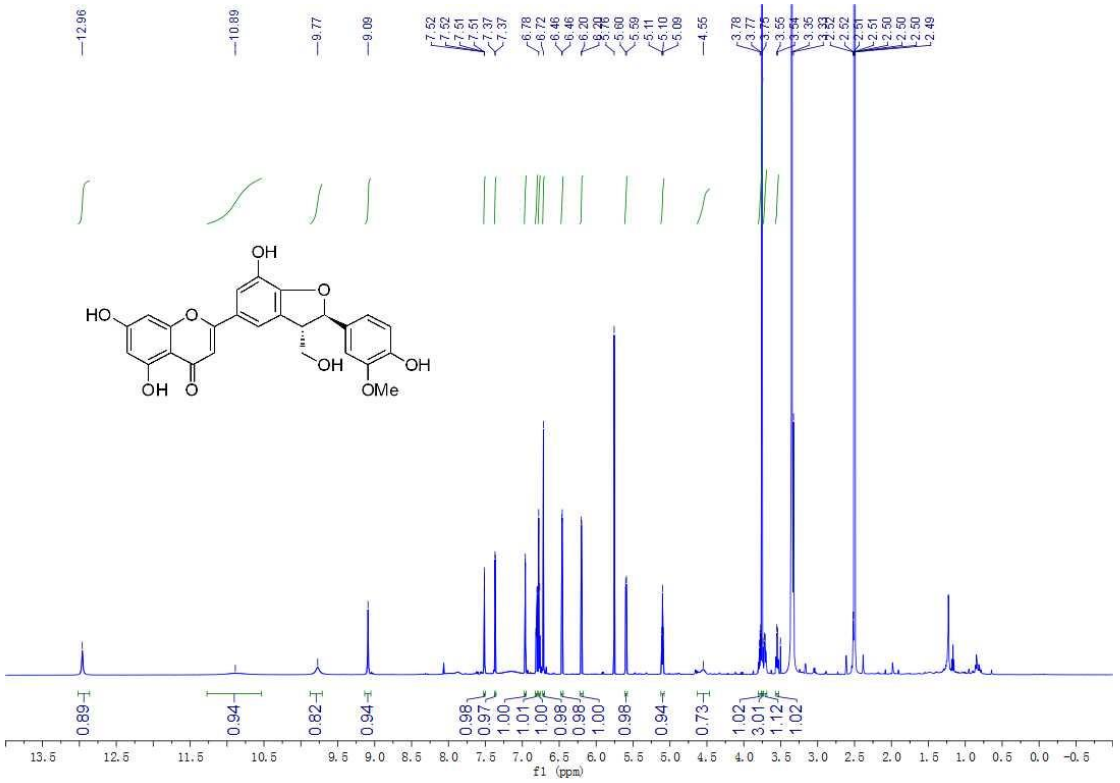

^{1}H NMR (600 MHz, DMSO-d_6) of **6**

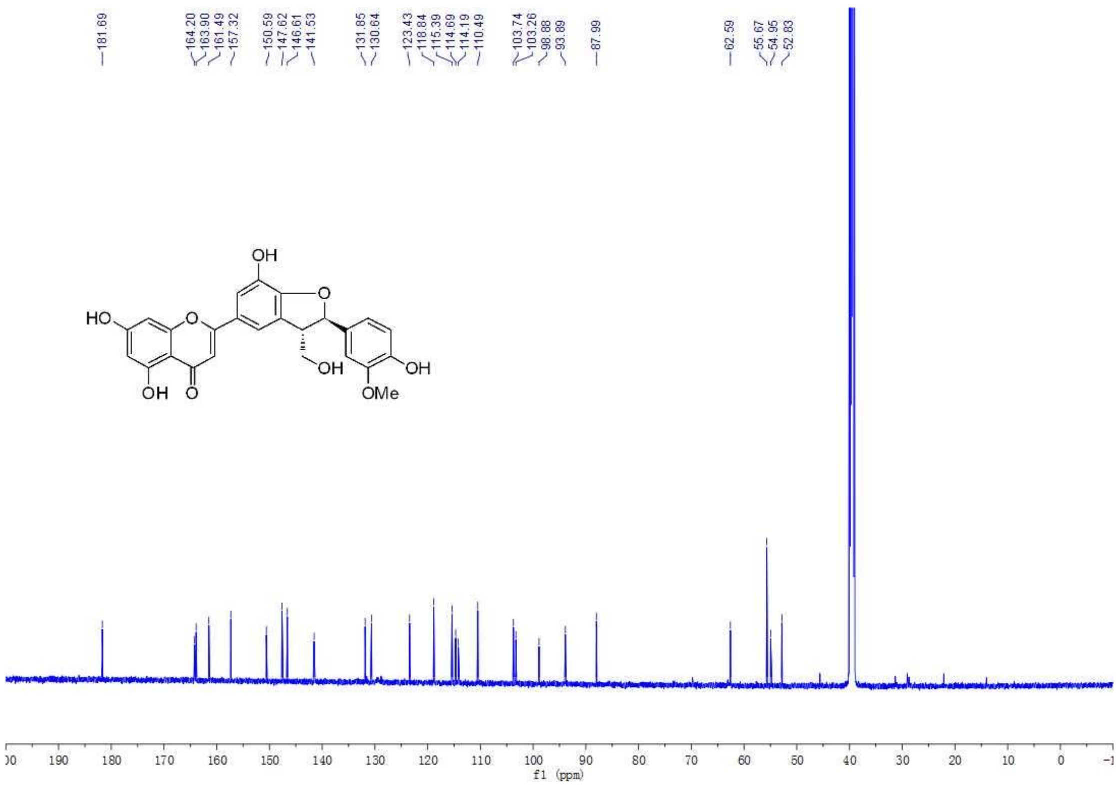

^{13}C NMR (151MHz, DMSO-d_6) of **6**
